# Final validation of the mental health screening tool for depressive disorders: A brief online and offline screening tool for major depressive disorder

**DOI:** 10.3389/fpsyg.2022.992068

**Published:** 2022-10-05

**Authors:** Kiho Park, Seowon Yoon, Surin Cho, Younyoung Choi, Seung-Hwan Lee, Kee-Hong Choi

**Affiliations:** ^1^School of Psychology, Korea University, Seoul, South Korea; ^2^Department of Psychology, Ajou University, Seoul, South Korea; ^3^Department of Psychiatry, Ilsan Paik Hospital, Inje University College of Medicine, Goyang, South Korea; ^4^KU Mind Health Institute, Korea University, Seoul, South Korea

**Keywords:** screening tests, depression, psychometrics, item response theory, diagnostic utility, online assessment

## Abstract

Early screening for depressive disorders is crucial given that major depressive disorder (MDD) is one of the main reasons of global burden of disease, and depression is the underlying cause for 60% of suicides. The need for an accurate screening for depression with high diagnostic sensitivity and specificity in a brief and culturally adapted manner has emerged. This study reports the final stage of a 3-year research project for the development of depression screening tool. The developed Mental Health Screening Tool for Depressive Disorders (MHS:D) was designed to be administered in both online and offline environments with a high level of sensitivity and specificity in screening for major depressive disorder. A total of 527 individuals completed two versions (online/offline) of the MHS:D and existing depression scales, including the BDI-II, CES-D, and PHQ-9. The Mini International Neuropsychiatric Interview (MINI) for diagnostic sensitivity/specificity was also administered to all participants. Internal consistency, convergent validity, factor analysis, item response theory analysis, and receiver operating characteristics curve (ROC) analysis were performed. The MHS:D showed an excellent level of internal consistency and convergent validity as well as a one-factor model with a reasonable level of model fit. The MHS:D could screen for major depressive disorder accurately (0.911 sensitivity and 0.878 specificity for both online and paper-pencil versions). Item response theory analysis suggested that items from the MHS:D could provide significantly more information than other existing depression scales. These statistical analyses indicated that the MHS:D is a valid and reliable scale for screening Korean patients with MDD with high diagnostic sensitivity and specificity. Moreover, given that the MHS:D is a considerably brief scale that can be administered in either online or paper-pencil versions, it can be used effectively in various contexts, particularly during the pandemic.

## Introduction

Major depressive disorder (MDD) is one of the most common mental disorders. The Global Burden of Disease Study regards it as a leading cause of disease burden worldwide (Ferrari et al., 2013). In particular, Korea is suffering from high suicide rate, and depression is the cause of 60% of suicides (Jeon, 2011). Owing to the COVID-19 pandemic, reports of mild to moderate levels of depressive symptoms have rapidly increased. Prior to the pandemic, only 15.5% of people reported experiencing mild or higher levels of depressive symptoms. However, in September 2020, Ministry of Health and Welfare, Republic of Korea (MOHW) reported that 49.2% of Korean people reported mild or higher levels of depressive symptoms. As of June 2021, the rate slightly decreased to 42.07% (Ministry of Health and Welfare, Republic of Korea, 2021).

A significant problem is the fact that most people experiencing depression do not seek help from professional mental health services. According to the National Mental Health Survey in Korea, only 22% of people diagnosed with mental disorders reported using mental health services. The major reason for their failure to use mental health services (81%) was the lack of information available about their mental health status (Ministry of Health and Welfare, Republic of Korea, 2016). Globally, it is common for patients with depressive disorders to visit primary care settings for physical or somatic concerns, such as fatigue, poor concentration, insomnia, and changes in appetite, rather than for mental health issues (Zich et al., 1990). Previous meta-analysis study revealed that only 47.3% of patients with depression who visited primary care institutions were accurately diagnosed with depression (Mitchell et al., 2009). Thus, it is crucial to screen for depressive symptoms in diverse healthcare settings, including primary care (Pignone et al., 2002).

To accurately screen for depression from its early stages, the role of the screening tool is paramount. In Korea, screening tools developed in foreign countries are mainly used, including Beck Depression Inventory-II (BDI-II) (Beck et al., 1996), the Center for Epidemiologic Studies-Depression Scale (CES-D) (Radloff, 1977), and the Patient Health Questionnaire-9 (PHQ-9) (Kroenke et al., 2001; Park et al., 2010). Although these scales have been utilized to screen and measure the severity of depression in psychiatric areas for a long time, they have some limitations. For example, the BDI-II is considered more suitable as a severity measuring tool than a screening tool because of the length of the scale (Park et al., 2020). The biggest limitation of these scales, however, is that they were not developed in the Korean language with the Korean population in mind.

When adopting a foreign-developed screening tool in other cultural and language-based countries, there are many factors to consider. Research from Japan has reported that due to the tendency to suppress positivity, the General Health Questionnaire (Iwata et al., 1994) and CES-D (Iwata and Buka, 2002) both showed much lower positive emotion scores and a lack of emotion-related questions compared to Western research Furthermore, in the Vietnamese language, there are no words for psychiatry or depression (Phan and Silove, 1997). The absence of these words goes beyond just the problem of translation and suggests that the way Vietnamese people manifest depression may differ from the way western people do. Some studies suggested that Koreans are less likely to explicitly share positive feelings and emotions (Kim et al., 1992; Noh et al., 1992; Cho and Kim, 1998). Thus, when using one scale translated into different languages, despite accurate translation, people from different cultures may interpret it in different semantic ways. Second, the manifestation of depression and the concept to be measured can differ due to cultural difference. Third, even if the same construct concept is measured in the same sentence, psychometric properties and response patterns for each item will inevitably differ according to the cultural background (Iwata and Buka, 2002).

This research aims to develop a scale that can accurately and efficiently screen depressive disorder for Korean people. In the development process, we focused on two aspects. The first is to develop a scale based on Koreans’ item responses. To this end, the item response theory (IRT) was applied in addition to the classical test theory (CTT). The CTT assumes that the observed test score is the sum of the true score and the error score, which are independent of each other (Rusch et al., 2017). Although the CTT has been widely utilized in psychometric areas, it has several limitations. In the CTT, the ability score may change depending on the question; conversely, the characteristics of the scale, such as difficulty or discrimination, may vary depending on the research sample. The CTT also assumes the same measurement error for all subjects. However, the measurement error may vary depending on the ability level of the subjects, the purpose of the test, or for other reasons. These problems can be solved using the IRT. As the IRT estimates both item parameters and a person’s ability parameter and expresses them on the same standardized scale, the estimated item parameter does not change according to the subjects; the subjects’ ability parameters are not affected by the test. In addition, the significant advantage of the IRT in test development is that it provides measurement errors and information functions for each item. This information provides a sound basis for choosing items from a large item pool. The IRT has already been widely utilized in the field of education, and recently, psychometricians have begun to adopt this theory. For example, Gibbons et al. (2013) developed a computerized adaptive test (CAT) for depression screening based on the IRT and reported successful screening performance.

The second aspect is to develop a scale for use in paper-and pencil-based offline situations and online situations. Online screening for depression has been developing since the late 1990s (Ogles et al., 1998). In addition to the advantage of online screening, which can be conducted with a large population at a low cost (Houston et al., 2001; Drake et al., 2014), due to the COVID-19 pandemic, the ability to perform the test in non-contact situations has drawn more attention. Several studies have compared the psychometric properties of paper and online versions (Holländare et al., 2010; Cronly et al., 2018) and reported the equivalence of the results. However, previous research adopted traditional paper-based scales in an online environment; no screening tool has been designed for use in both online and offline environments from the development stage.

The purpose of this study is to develop an online/offline (paper-pencil) version of a depression screening tool suitable for the Korean population and evaluate its psychometric properties. Additionally, our screening scale aims to show higher sensitivity, specificity, positive predictiveness, and negative predictiveness compared to other existing screening tools.

## Materials and methods

### Development procedure

This study is part of a nationwide multi-site study aiming to develop Korean depression, anxiety, and suicidality screening scales (Yoon et al., 2018, 2020; Kim et al., 2021). The Mental Health Screening Tool for Depressive Disorders (MHS:D) was developed in three stages over 3 years (2016–2018). The detailed procedure followed in its development is presented below. Details regarding stage 1 are covered by Jung et al. (2017) and those regarding stage 2 are covered by Yoon et al. (2018).

In the first stage (stage 1), a preliminary item pool with 383 items was developed (Jung et al., 2017). Items were collected through a literature review and focus group interviews. A literature review of 23 widely used self-report questionnaires relating to depressive disorders, bipolar disorders, and suicidality was performed. Focus group interviews were conducted with seven MDD patients diagnosed by a psychiatrist. Interviews were conducted by a licensed psychologist and two clinical psychology graduate students. Further, the interviews were recorded, and the research team of three licensed clinical psychologists, a psychiatrist, and a psychometric expert derived unique items from the interviews. The tense of the items was determined as “the last 2 weeks” following the diagnostic criteria of the DSM-5. Reverse wording items were excluded because of their ineffectiveness (Van Sonderen et al., 2013). Further, items measuring domain (e.g., depressed mood, loss of interest) and item difficulty (e.g., “Sometimes I feel depressed” was coded as “mild” and “I am depressed all the time” was coded “severe”) were coded so that the item pool could cover various domains and difficulties. A total of 383 items were tested in a sample of 153 non-clinical participants and 105 patients with MDD. Using the CTT and the IRT, we analyzed the results and selected 170 items that accurately discriminated patients with MDD from non-clinical participants (Jung et al., 2017).

The second stage (stage 2) study was conducted in 2018, with a sample of 613 participants responding to the 170 items selected in the stage 1 (Yoon et al., 2018). Other depression scales such as the BDI-II, PHQ-9, CES-D, and Generalized Anxiety Measuring Scale (GAD-7) were tested together to confirm convergent and discriminant validity. Finally, the Mini-International Neuropsychiatric Interview (MINI) Plus version 5.0.0 was conducted for psychiatric diagnosis. The interviews were conducted by trained interviewers, and diagnostic decisions were made through case conferences with licensed psychologists and a psychiatrist. All interviewers were blinded to the results of self-report questionnaires. After analysis with CTT and IRT, we developed a combination of 12 items that best discriminated depressive participants from non-depressive participants (Yoon et al., 2018).

**Figure 1:**
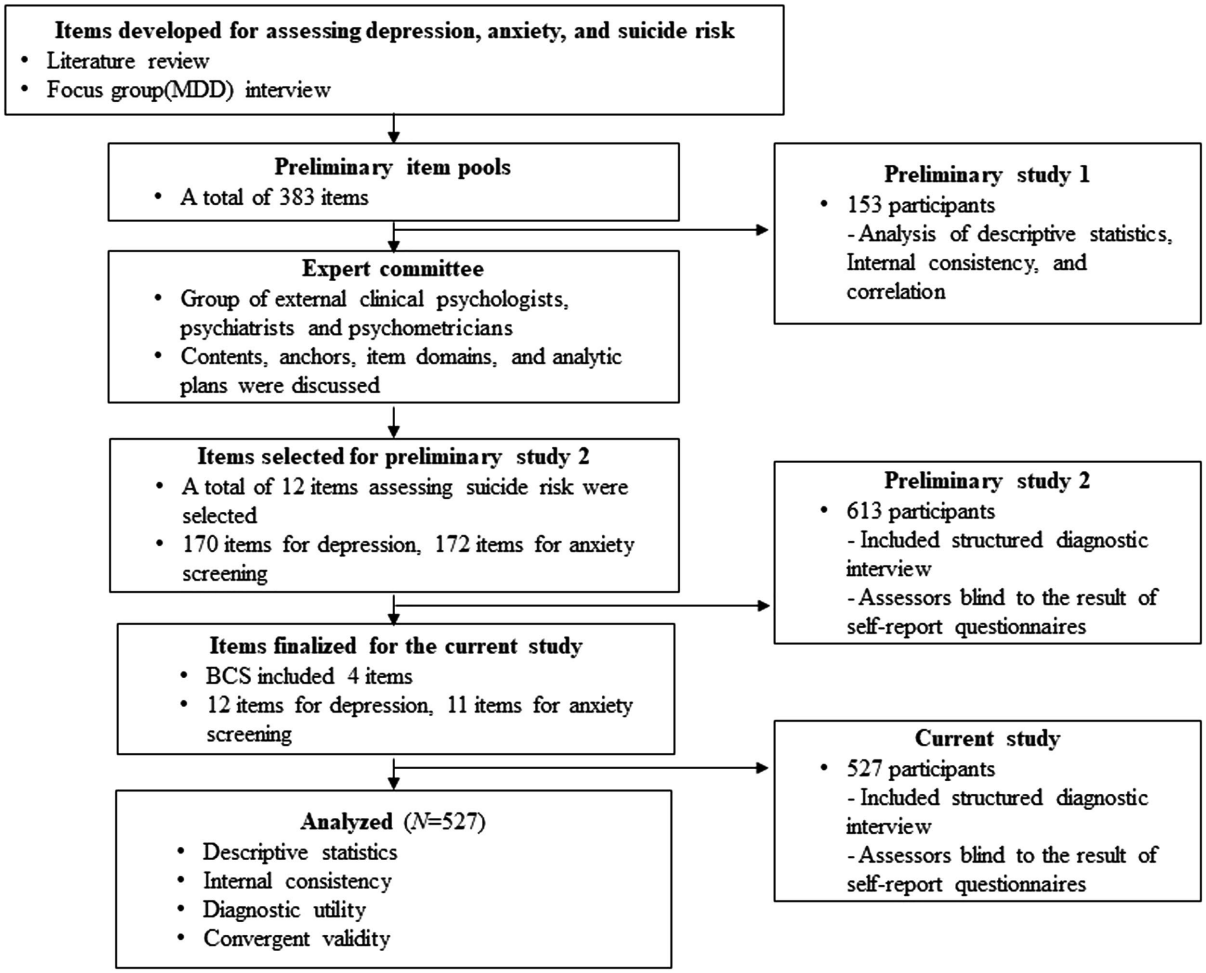
Development procedure.

The current study (stage 3) examines the 12 MHS:D items finalized from the previous stages. The validation process of the current study includes examining psychometric properties and diagnostic accuracy. The BDI-II, PHQ-9, CES-D, and GAD-7 were conducted along with the final version of the MHS:D to examine convergent and discriminant validity; the MINI Plus version 5.0.0. was used for the psychiatric diagnosis to examine criterion validity. Trained interviewers conducted the structured clinical interview while being blinded to the results of the self-report questionnaires. Two licensed psychologists and a psychiatrist supervised the interviews, and diagnostic decisions were made through case conferences. Detailed developmental process is depicted in Figure 1.

### Participants

A total of 527 participants completed both online and offline versions of MHS:D. Of the 527 participants, 257 were recruited *via* an online advertisement, and the rest 270 participants were recruited from college hospital visitors using a consecutive sampling method. The number of the participants of the current study was considered sufficient for conducting ROC (Receiver Operating Characteristics) analysis since Bujang and Adnan (2016) suggested that a sample of minimum 300 subjects is sufficiently large for to evaluate sensitivity and specificity of diagnostic tests. The participants from the hospitals included clinical (e.g., psychiatric or non-psychiatric) and healthy samples. Equivalently, the participants recruited from the online advertisement included either clinical or healthy samples as well. The only inclusion criterion of the current study was being over 18 years. The exclusion criteria were as follows: (1) inappropriate responses, (2) history of neurological surgery (e.g., brain surgery), (3) presence of other severe disorders that significantly disturbed test administration, and (4) aged below 19 years. All participants participated voluntarily and signed written informed consent forms. The study participants were provided with a remuneration of 10,000 KRW (approximately 10 USD). The Institutional Review Boards of Korea University (1040548-KU-IRB-15-92-A-1(R-A-1)(R-A-2)(R-A-2)) and the Ilsan Paik Hospital (ISPAIK 2015–05–221-009) approved the study. Detailed demographic information is presented in Table 1.

**Table 1:** Sample demographics.

	Total sample (*N* = 527)
	*M* (*SD*)
Age	38.6 (15.0)
Education (years)	14.6 (3.2)
	*N* (%)
*Gender*	
Female	340 (64.5)
*Marital status*	
Single	285 (54.1)
Married	214 (40.6)
Divorced	8 (1.5)
Widowed	10 (1.9)
Unreported	10 (1.9)

### Measures

#### Mental health screening tool for depressive disorders

The MHS:D is a depression screening tool with 12 items and covers all nine criteria for the diagnosis of MDD from the DSM-5 (depressed mood, loss of interest, psychomotor agitation, change in appetite, sleep disturbance, fatigue, concentration difficulty, feeling worthless, thoughts of suicide). As the appetite-related items are separated into two items (increased and decreased appetite), a total of 10 items were derived from the DSM-5 diagnostic criteria. Two items that measure helplessness and hopelessness, which, in the preliminary examination, were found to effectively screen Koreans’ depression, were added to the test, and 12 items were developed. Each item was scored on a 5-point Likert scale: 0 (not at all), 1 (slightly), 2 (moderately), 3 (very), 4 (extremely).

When scoring the test, the weight of each item derived from IRT analysis was multiplied by the response to each item, and the values were summed. In the calculation process, the appetite increase/decrease item was converted into one value with the highest score among the two items. Therefore, the value of 11 items was utilized for the final score, and the statistical analysis in the results section is also based on the 11 items.

#### MINI-international neuropsychiatric interview plus version 5.0.0

The MINI (Sheehan et al., 1998) is a structured clinical interview developed to screen for mental disorders. Each mental disorder diagnosis was based on the 10th edition of the International Classification of Diseases (ICD-10) and the 4th edition of the Diagnostic and Statistical Manual of Mental Disorder (American Psychiatric Association, 2013). This study utilized all modules of the MINI, and the depression module was utilized as a reference standard for the presence of depressive disorders. Other modules of the MINI that were utilized in this study include bipolar disorders, anxiety disorders, schizophrenia spectrum disorders, substance use disorders, and obsessive–compulsive disorders. This study adopted the Korean version of the MINI, which has an adequate level of diagnostic accuracy (Yoo et al., 2006); The intraclass correlation coefficient (ICC) as a measure of inter-rater reliability for the MINI diagnoses was 0.92 in the current study.

#### Beck depression inventory-II

The BDI-II is a measure that assesses depressive symptoms using 21 items on a 4-point Likert scale (Beck et al., 1996). Each item of the BDI-II measures emotional, cognitive, motivational, and physiological domains of depression, with scores ranging from 0 to 3. BDI-II scores of 0–13 indicate minimal depression, 14–19 indicate mild depression, 20–28 indicate moderate depression, and 29–63 indicate severe depression. This study used the Korean version of the translated by Lee et al. (2017) and validated by Park et al. (2020). Cronbach’s alpha coefficient for internal consistency of the Korean version of the BDI-II was 0.946, a cut-off point of 23 with a sensitivity of 0.833, a specificity of 0.868 and AUC of 0.915 (Park et al., 2020).

#### Patient health questionnaire-9

The PHQ-9, developed by Kroenke et al. (2001), measures depressive symptoms, including depression, sleep and appetite changes, unpleasantness, fatigue, inappropriate guilt, unreasonableness, loss of concentration, and suicidal thoughts. Each item is scored on a 4-point Likert scale ranging from 0 (not at all) to 3 (almost daily). Higher scores on the PHQ-9 indicate more severe depressive symptoms. This study adopted the Korean version of the PHQ-9, developed by Park et al. (2010). This version possesses an adequate level of reliability with a Cronbach’s alpha of 0.81 and test–retest reliability of 0.89 (Park et al., 2010).

#### Center for epidemiological studies depression scale

The CES-D is a self-administered 20-item scale that assesses the frequency of depressive symptoms (Radloff, 1977). The CES-D items include questions related to depressive mood, helplessness, hopelessness, appetite change, sleep disturbance, and inappropriate feelings of guilt. The respondents were asked to choose one of four answers that best described the frequency of their depressive symptoms. The 4-point Likert scale of the CES-D ranges from 0 (none of the time) to 3 (most or all of the time). This study adopted the Korean version of the CES-D translated and validated by Cho and Kim (1998). The Korean version of the CES-D possesses an adequate level of psychometric properties with a Cronbach’s alpha of 0.89, test–retest Pearson’s correlation of 0.68, and a cut-off point of 25 with a sensitivity of 0.91 and specificity of 0.78.

#### Generalized anxiety disorder 7-item scale

The GAD-7 (Spitzer et al., 2006), a screening tool for generalized anxiety disorder, measures the severity of anxiety symptoms. The respondents were asked how frequently they had experienced anxiety symptoms during the past 2 weeks. The 4-point Likert scale of the GAD-7 ranged from 0 to 3. The Korean version of the GAD-7 was adopted in this study, and its validity has been examined by Ahn et al. (2019). Cronbach’s alpha coefficient for internal consistency of the Korean version of the GAD-7 was 0.93, a cut-off point of 8 with a sensitivity of 0.81, a specificity of 0.85 and AUC of 0.91 (Ahn et al., 2019).

### Statistical analysis

The IBM SPSS Statistics 25 program was used for descriptive statistics, correlational analysis, and receiver operating characteristic (ROC) curve analysis. The R statistical program (version 3.5.0) was used to perform the factor analysis and IRT analysis. For factor analysis, the “Lavaan” package (Rosseel et al., 2017) was utilized. The estimation was conducted using the maximum-likelihood method. Incremental fit indices and absolute fit indices were used to evaluate the model fit. Incremental fit indices included the Tucker Lewis Index (TLI) and comparative fit index (CFI). For absolute model fit, the root mean square error of approximation (RMSEA) and the standardized root mean squared residual (SRMR) were included. Interpretation of model fit indices followed standard criteria (CFI and TLI > 0.90 and RMSEA and SRMR <0.08) (Hooper et al., 2008). For IRT analysis, the “mirt” package (Chalmers, 2012) was utilized. When performing the analysis, a graded response model (GRM) appropriate for ordered polytomous categories such as Likert scales (Samejima, 1997) was adopted.

## Results

### Item characteristics

The average of unweighted MHS:D total scores for all participants was 9.12 (*SD* = 10.01) for the paper-pencil version and 9.07 (*SD* = 9.70) for the online version. Using MINI psychiatry structured interviews, 58 participants were diagnosed with MDD. Unweighted total scores on the MHS:D for MDD patients were 28.05 (*SD* = 9.16) for the paper-pencil version and 26.78 (*SD*: 9.73) for the online version. The non-MDD sample showed 6.81 (*SD* = 7.30) for the paper-pencil version and 6.88 (*SD* = 7.12) for the online version. As we did not recruit healthy and MDD patients separately but randomly recruited samples and then conducted diagnostic interviews, the non-MDD sample included not only a healthy population but also other psychiatric disorder patients and subthreshold depressive patients. The detailed means and standard deviations for each item are presented in Table 2.

**Table 2:** Mean, standard deviations, and item-total correlations of MHS:D.

No.	Item	Total sample		Non-MDD sample^a^		MDD patients		Cronbach’s alpha if item deleted		Item-total correlation	
		Paper-pencil version (*N* ^b^ = 524) mean (sd)	Online versio*n* (*N* = 527) mean (sd)	Paper-pencil versio*n* (*N* = 467) mean (sd)	Online versio*n* (*N* = 469) mean (sd)	Paper-pencil versio*n* (*N* = 57) mean (sd)	Online versio*n* (*N* = 58) mean (sd)	Paper-pencil version	Online version	Paper-pencil version	Online version
1	Depressed mood	0.79 (1.06)	0.83 (1.08)	0.57 (0.82)	0.63 (0.86)	2.61 (1.03)	2.52 (1.14)	0.935	0.937	0.870^***^	0.875^***^
2	Loss of interest	0.98 (1.10)	0.99 (1.09)	0.78 (0.92)	0.81 (0.93)	2.65 (1.08)	2.48 (1.11)	0.936	0.939	0.839^***^	0.829^***^
3	Psychomotor agitation	0.94 (1.11)	0.99 (1.14)	0.72 (0.91)	0.77 (0.94)	2.72 (0.97)	2.71 (1.17)	0.936	0.938	0.846^***^	0.843^***^
4	Fatigue	0.77 (1.16)	0.78 (1.12)	0.55 (0.96)	0.57 (0.91)	2.54 (2.0)	2.50 (1.23)	0.937	0.938	0.820^***^	0.845^***^
5	Feeling worthless	0.65 (1.12)	0.63 (1.09)	0.41 (0.82)	0.42 (0.81)	2.67 (1.19)	2.38 (1.32)	0.935	0.937	0.858^***^	0.872^***^
6	Concentration difficulty	0.98 (1.17)	0.89 (1.07)	0.77 (1)	0.7 (0.89)	2.68 (1.11)	2.43 (1.13)	0.939	0.941	0.790^***^	0.786^***^
7	Thoughts of suicide	0.47 (0.94)	0.45 (0.90)	0.28 (0.70)	0.27 (0.63)	1.96 (1.23)	1.93 (1.31)	0.938	0.94	0.799^***^	0.808^***^
8	Helplessness	0.55 (1.03)	0.52 (0.99)	0.35 (0.79)	0.31 (0.71)	2.21 (1.25)	2.21 (1.31)	0.936	0.939	0.837^***^	0.832^***^
9	Hopelessness	0.69 (1.12)	0.69 (1.05)	0.48 (0.88)	0.48 (0.81)	2.49 (1.27)	2.40 (1.24)	0.937	0.938	0.821^***^	0.837^***^
10/11	Increased/decreased appetite	1.12 (1.25)	1.13 (1.17)	0.95 (1.15)	0.98 (1.07)	2.51 (1.23)	2.38 (1.21)	0.945	0.947	0.674^***^	0.673^***^
12	Sleep disturbance	1.10 (1.31)	1.10 (1.27)	0.91 (1.16)	0.91 (1.14)	2.72 (1.40)	2.64 (1.28)	0.944	0.947	0.701^***^	0.698^***^
	Total item	9.12 (10.01)	9.07 (9.70)	6.81 (7.30)	6.88 (7.12)	28.05 (9.16)	26.78 (9.73)	0.935	0.937	0.913^***^ ^a^	

*** *p* < 0.001.

a Correlation between offline version total score and online version total score.

b The number of participants who completed paper-pencil version of MHS:D may be < 527 due to missing data.

### Internal consistency and convergent validity

Cronbach’s alpha coefficient for the MHS:D was 0.94 for the paper-pencil version and 0.95 for the online version, which indicates a high level of internal consistency. No item was suggested to be excluded from the test to enhance internal consistency. Detailed Cronbach’s alpha coefficients if individual items deleted are shown in Table 2.

Item-total correlation ranged from.67 to 0.87 for the paper-pencil version and from 0.67 to 0.88 for the online version. The items with the highest and lowest correlation were “depressed mood” and “sleep disturbance,” respectively, for both paper-pencil and online versions. The correlational coefficients between each item and the total score are presented in Table 2.

To examine convergent validity, a correlational analysis with existing depression measurements (CES-D, BDI-II, and PHQ-9) was conducted. Correlational coefficients ranged from 0.85 to 0.89, which indicates a high level of convergent validity. The MHS:D also showed a high correlation between the GAD-7 total score—a screening tool for generalized anxiety (GAD)— and is frequently comorbid with depression. The detailed correlation coefficients are listed in Table 3.

**Table 3:** Correlation coefficients between MHS:D and related measures.

	CES-D	PHQ-9	BDI-II	GAD-7
Paper-pencil MHS:D	0.878^**^	0.889^**^	0.863^**^	0.839^**^
Online MHS:D	0.852^**^	0.865^**^	0.849^**^	0.807^**^

**p < 0.01.

### Factor structure

Exploratory factor analysis (EFA) and confirmatory factor analysis (CFA) were performed to identify and confirm the factor structure of the MHS:D. Both paper-pencil and online data were randomly split in half to perform two different analyses. The principal axis factoring method was applied to perform EFA. The results of EFA suggested a one-factor model for both the paper-pencil and online versions. The total explained variance is presented in Table 4, and the scree plot is presented in Supplementary Figure 1.

**Table 4:** Total explained variance for offline and online version of MHS:D.

Factor	Paper-pencil version		Online version	
	Initial eigenvalues total	Initial eigenvalues percent of variance	Initial eigenvalues total	Initial eigenvalues percent of variance
1	7.360	66.906	7.194	65.404
2	0.752	6.837	0.857	7.795
3	0.576	5.233	0.647	5.885
4	0.537	4.882	0.447	4.061
5	0.433	3.937	0.418	3.798
6	0.355	3.229	0.332	3.020
7	0.303	2.753	0.325	2.956
8	0.221	2.005	0.248	2.256
9	0.172	1.565	0.219	1.992
10	0.163	1.479	0.191	1.739
11	0.129	1.174	0.120	1.094

CFA was performed to confirm the one-factor model with the remaining half of the data. The exploratory structural equation modeling (ESEM) method was also applied to the traditional CFA method as recommended by Marsh et al. (2009). The inspection of modification indices (MI) suggested that the inclusion of two correlated residuals (items 1 and 2 and items 5 and 9) would improve the model fit substantially for both the paper-pencil and online versions of the MHS:D. Items 1 and 2 measure feelings of depression and loss of interest, respectively, and these two domains are essential symptoms for diagnosing depressive disorders. Item 5 is about worthlessness, item 9 is related to hopelessness, and both are semantically similar—they are negative evaluations of one’s current life and future. Based on MI and semantic similarity, the correlation between the residuals of items 1 and 2 and items 5 and 9 were added to the model. A summary of goodness-of-fit indices for CFA is presented in Table 5, and the factor structure is presented in Supplementary Figure 2. The result of the one-factor factor analysis of the MHS:D showed reasonable model fit indices for both the online and paper-pencil versions. Both TLI and CFI reached the criteria of over 0.90. For absolute model fit, the criterion for the SRMR, which is below 0.05, was satisfied. However, the criterion for the RMSEA was not satisfied. Information indices were not interpreted as there were no other models for comparison.

**Table 5:** Summary of goodness-of-fit indices for CFA.

	Fit indices								
Model tested	*χ^2^*	AIC	BIC	aBIC	CFI	TLI	SRMR	RMSEA	90% CI
Paper-pencil version	162.244^***^ (*d*f = 42)	6650.791	6736.432	6660.341	0.945	0.928	0.042	0.105	0.088–0.122
Online version	227.508^***^ (*d*f = 42)	6043.834	6129.657	6053.565	0.928	0.906	0.047	0.129	0.113–0.146

***p < 0.001.

### Item response theory analysis

To evaluate suitability and relevancy, a polytomous item response theory analysis was performed from the developmental stage. The graded response model suggested by Samejima (1997) was used for the analysis. The item parameters for each item are listed in Table 6. Item characteristic curves for each item are presented in Supplementary Figure 3 for the paper-pencil version and Supplementary Figure 4 for the online version.

**Table 6:** Item parameters and weight.

	Paper-pencil version						Online version					
	a	b1	b2	b3	b4	Weight	a	b1	b2	b3	b4	Weight
Item 1	4.24	0.04	0.95	1.39	2.08	1.12	4.09	−0.06	1.00	1.45	1.95	1.09
Item 2	3.57	−0.23	0.70	1.36	1.98	0.95	3.21	−0.31	0.78	1.41	2.06	0.99
Item 3	3.28	−0.15	0.77	1.39	2.00	1.00	3.00	−0.22	0.78	1.39	1.92	0.97
Item 4	2.81	0.26	0.89	1.40	2.02	1.14	2.92	0.14	1.04	1.50	1.96	1.16
Item 5	3.75	0.41	0.99	1.42	1.92	1.19	4.54	0.37	1.08	1.47	1.96	1.22
Item 6	2.28	−0.14	0.75	1.35	2.15	1.03	2.35	−0.15	0.92	1.57	2.43	1.19
Item 7	3.37	0.69	1.31	1.80	2.20	1.50	3.61	0.65	1.41	1.82	2.29	1.54
Item 8	3.58	0.58	1.13	1.54	2.23	1.37	3.88	0.59	1.21	1.58	2.14	1.38
Item 9	2.97	0.32	1.08	1.46	2.03	1.22	3.28	0.22	1.12	1.65	2.05	1.26
Items 10 and 11	1.50	−0.36	0.81	1.39	2.26	1.03	1.40	−0.57	0.80	1.73	2.50	1.12
Item 12	1.60	−0.22	0.78	1.30	1.93	0.95	1.54	−0.27	0.76	1.45	2.10	1.01

The results of the analysis showed that the boundary (difficulty) parameters (b1–b4 in Table 6) for each item are distributed appropriately without overlapping or transposition, which means that a separate Likert scale of 5 points possesses its own information. The weight of each item was calculated as the average of the difficulty parameters of each item. Item weight ranged from 0.95 to 1.50 for the paper-pencil version and from 0.97 to 1.54 for the online version. The item with the highest weight—an item that measures the most severe depressive symptom—was “thoughts of suicide.” The item discrimination parameter (“a” from Table 6) ranged from 1.50 to 4.24 for the paper-pencil version and 1.40 to 4.54 for the online version. The average discrimination parameter for all items was 3.00 for the paper-pencil version and 3.07 for the online version, respectively, indicating considerable test precision. The information value for each ability area (θ) and the total information value of the individual items are presented in Supplementary Table 1. Item information curves (IIC) for each item are presented in Supplementary Figure 5 for the paper-pencil version and Supplementary Figure 6 for the online version. Information values for the entire test are also presented in Supplementary Table 1, and the test information curves (TIC) are depicted in Supplementary Figure 7. The TICs of the MHS:D draw curves with a peak around 1.5 theta and provide the most information around the 1.0 to 2.0 theta area. The total amount of test information was also compared to other depression scales. Detailed test information values from these scales are presented in Supplementary Table 2. Both versions of the MHS:D showed similar amounts of test information compared to the BDI-II and the CES-D and 1.5 times bigger than the PHQ-9. Considering that the test information value is a simple summation of each item’s information value, it is more meaningful to compare the average amount of information for each item. Both versions of the MHS:D showed much higher information value for each item compared to other traditional depression scales.

### Analysis based on diagnosis

**Figure 2:**
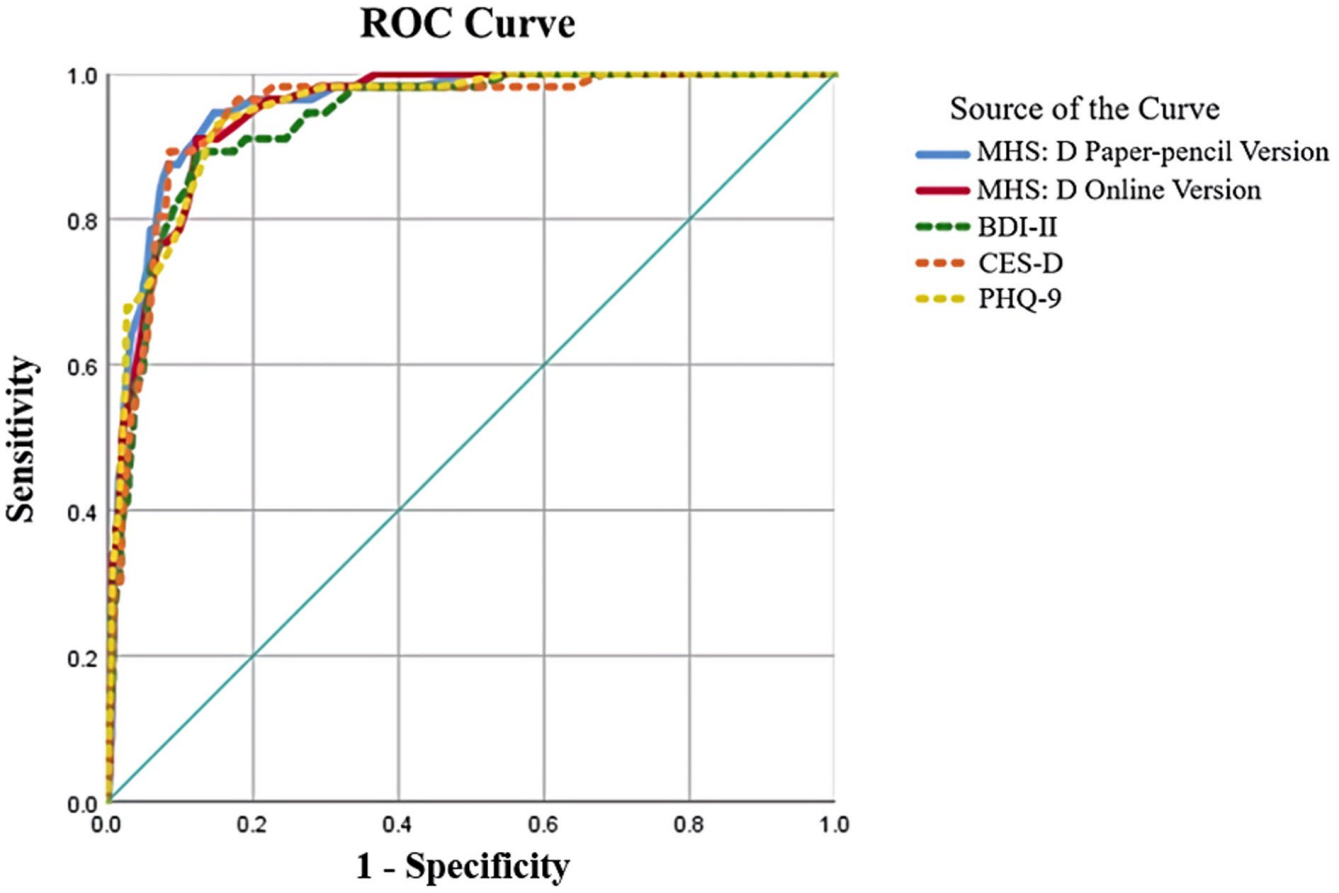
ROC curve for MHS:D and existing depression measure.

ROC curve analyses were conducted to identify the screening ability of the MHS:D. Weighted scores were adopted for this analysis. The detailed results of the analysis are presented in Table 7, and the ROC curves are depicted in Figure 2. To compare the ability to screen for MDD, an analysis was also conducted with existing depression scales such as the BDI-II, PHQ-9, and CES-D. The area under the curve (AUC) was 0.95 for the paper-pencil version and the online version. The optimal cut-off point was calculated using Youden’s index (Youden, 1950), and 17 points were selected for both the paper-pencil and online versions of the MHS:D’s cut-off point. The paper-pencil and online MHS:D showed 0.91 sensitivity and 0.88 specificity with the optimal cut-off score, manifesting better diagnostic accuracy in MDD screening compared to other existing depression scales.

**Table 7:** Results of ROC analyses for the MDD.

Measures and cut-off score	AUC	SEN	SPE	PPV	NPV
*Paper-pencil MHS:D (Cut-off = 17)*	0.953	0.911	0.878	0.477	0.988
*Online MHS:D (Cut-off = 17)*	0.947	0.911	0.878	0.482	0.988
BDI-II Mild = 14	0.935	0.982	0.629	0.247	0.997
BDI-II Moderate = 20		0.911	0.810	0.374	0.987
BDI-II Severe = 29		0.732	0.941	0.609	0.967
CES-D Mild = 16	0.945	0.982	0.533	0.208	0.996
CES-D Moderate = 28		0.911	0.860	0.445	0.988
PHQ-9 Mild = 5	0.947	0.982	0.541	0.210	0.996
PHQ-9 Moderate = 10		0.929	0.854	0.439	0.990
PHQ-9 Moderately severe = 15		0.696	0.954	0.645	0.961
PHQ-9 Severe = 20		0.411	0.985	0.759	0.930

AUC, area under the curve; SEN, sensitivity; SPE, specificity; PPV, positive predictive value; NPV, negative predictive value.

## Discussion

This research was the last stage of a mental health screening tool development project. To validate the final version of the MHS:D, the psychometric properties and diagnostic ability were examined. The results of the study showed that both the paper-pencil and online versions of the MHS:D are psychometrically sound and screened for MDD effectively.

Demographic and descriptive statistics showed that participants who were diagnosed with MDD reported significantly higher scores than the non-MDD sample. Non-MDD participants answered all items between “not at all” and “slightly”; patients with MDD answered between “moderately” to “very,” except for the “thoughts of suicide” item. The item that showed the biggest difference between two groups was “feeling worthlessness,” and the item which showed the smallest difference was “increased/decreased appetite” for both the paper-pencil and online versions. Considering the average standard deviation for each item, it is possible to say that non-MDD participants and patients with MDD reported significant differences.

The MHS:D also showed excellent internal consistency, convergent validity, and acceptable factor structure. The results of EFA strongly recommended the one-factor model solely, and this one-factor model was confirmed with CFA. All model fit indices, except for RMSEA, were satisfied, including CFI, TLI, and SRMR. In the structural equation model, however, it is considerably common for fit indices to be inconsistent (Lai and Green, 2016). Shi et al. (2020) conducted simulations for various conditions; across all simulated conditions, SRMR presented more reliable results than RMSEA. Therefore, the current study concluded that both the paper-pencil and online versions of the MHS:D fit well with the one-factor model since the MHS:D’s SRMR satisfied the recommended criterion.

From the developmental stage, IRT was adopted and played an important role in choosing items from the item pool. Consequently, all items’ ICC were distributed adequately, except for appetite and sleep-related items. In fact, appetite and sleep-related items are constantly reported as having low item information and poor ICC shape. Therefore, there was considerable speculation in the research team as to whether to include these items. The advisory group, which mainly comprised clinical experts, strongly recommended including those items, insisting on the importance of appetite and sleep-related problems in the clinical field. Even if the two items were included, their diagnostic ability was not significantly impaired. Therefore, these two items were included in the final product of the MHS:D despite relatively low item information. Nevertheless, the MHS:D could provide a similar level of information with approximately half the number of items in BDI-II or CES-D and provide much higher information than PHQ-9. Moreover, the current study validated both online and offline versions of MHS:D. Herman (2006) suggested that an effective screening test should be inexpensive and easy to administer, with minimal discomfort and morbidity to the participant. The importance and clinical significance of online mental health assessment has increased since COVID-19, because online mental health screening tools can reduce costs, enable efficient data collection, and improve convenience (Martin-Key et al., 2022). However, previous depression assessment tools have barely compared their online version with offline version which can differ. The current study confirmed that there was no psychometric difference between online version and offline version of the MHS:D.

Furthermore, our research team previously suggested that the Korean version of the BDI-II’s TIC shaped plateau-like curve indicates that the BDI-II is more suitable as a severity measuring tool than as a screening (Park et al., 2020). The MHS:D formed curves with a peak, which suggests appropriateness as a screening tool.

ROC curve analysis was conducted to examine the final diagnostic accuracy of the MHS:D. As mentioned above, the weight score induced by IRT analysis was applied when conducting the ROC analysis. The MHS:D produced an excellent AUC value. The MHS:D showed the highest or at least equivalent level of AUC compared to other depression measures. Additionally, the MHS:D produced the best level of Youden’s index (sensitivity + specificity – 1), which is a method for obtaining thresholds on medical tests while maintaining the highest level of positive predictive value. The results of ROC curve analysis suggest that both the paper-pencil and the online version of the MHS:D have an excellent level of screening for depression in the Korean population.

Some limitations should be noted in future studies. First, we used a consecutive sampling method instead of random sampling. The majority of the sample resided near Seoul and the capital area. Female participants almost doubled male participants in number. However, the result of IRT analysis indicated there was no significant difference in response patterns between gender. Male, adolescent, and geriatric samples are required for wider use. Second, the test–retest reliability was not reported in this study. To ensure the reliability and stability of the scores over time, a test–retest should be reported in future studies. Third, the result of the correlation analysis between MHS:D and GAD-7 was higher than expectation, with the correlation coefficient of higher than 0.8. However, some previous studies reported strong association between generalized anxiety disorder and depressive symptoms (Spitzer et al., 2006; Löwe et al., 2008; Seo et al., 2014). Brown et al. (2001) and Watson (2005) also noted that GAD is closely related to depressive disorders. Considering this, it seems to support the high correlation between MHS:D and GAD-7. Hence, in the case of respondents who have high scores or report anxiety-related symptoms while using MHS:D, it is recommended to proceed with a search related to GAD.

Despite the aforementioned limitations, the MHS:D is a reliable, valid, and highly efficient screening tool for MDD. As it is designed based on the item response patterns of Koreans, the MHS:D can provide a significant amount of information for clinicians with a few items. Moreover, MHS:D can be easily adopted by practitioners since it does not require specific qualifications on its use. The diagnostic accuracy of the MHS:D is expected to help screen depression patients in the early stages and ensure intervention, which will relieve the substantial economic social burden in Korea suffering from high suicide rate (World Health Organization, 2018).

Additionally, one of the most unique aspects of the MHS:D is that it is developed and validated on both online and paper-pencil platforms. Recently, because of the pandemic situation, the demand for psychological services has grown, while visiting hospitals or counseling centers has become more difficult because of the risk of infection. Non-contact based online medical services are attracting increasing attention. In this scenario, the MHS:D, which is available in both online and offline environments, should be considered as a useful screening tool for MDD.

## Data availability statement

The raw data supporting the conclusions of this article will be made available by the authors, without undue reservation.

## Ethics statement

The studies involving human participants were reviewed and approved by Korea university institutional review board. The patients/participants provided their written informed consent to participate in this study.

## Author contributions

KP, YC, S-HL, and K-HC devised the study, main conceptual ideas, and the study process. K-HC supervised the overall study process and direction. KP, SY, and SC contributed to the data collection, methodology, and the writing of the manuscript. K-HC reviewed and supervised the drafting of the manuscript. All authors contributed to the article and approved the submitted version.

## Funding

This research was supported by the Ministry of Education of the Republic of the Korea and National Research Foundation of Korea (NRF-2017S1A5B6053101), a grant of the Korea Health Technology R&D Project through the Korea Health Industry Development Institute (KHIDI), funded by the Ministry of Health & Welfare, Republic of Korea (grant number: HI21C0268), and the Korea Mental Health Technology R&D Project under the Korean Ministry of Health and Welfare (grant number: HM15C1169).

## Conflict of interest

The authors declare that the research was conducted in the absence of any commercial or financial relationships that could be construed as a potential conflict of interest.

## Publisher’s note

All claims expressed in this article are solely those of the authors and do not necessarily represent those of their affiliated organizations, or those of the publisher, the editors and the reviewers. Any product that may be evaluated in this article, or claim that may be made by its manufacturer, is not guaranteed or endorsed by the publisher.
