# Supplementary material for: Final validation of the mental health screening tool for depressive disorders: A brief online and offline screening tool for major depressive disorder
Source: Front Psychol. 2022 Oct 5;13:992068. doi: 10.3389/fpsyg.2022.992068 (PMC9580402; doi:10.3389/fpsyg.2022.992068)
Supplement: Supplementary file 1 [file Table_1.docx]

**Supplementary Table 1. Item & Test Information Value**

|  | Offline Version | | | | | | Online Version | | | | | |
| --- | --- | --- | --- | --- | --- | --- | --- | --- | --- | --- | --- | --- |
|  | -2≤θ<-1 | -1≤θ<0 | 0≤θ<1 | 1≤θ<2 | 2≤θ<3 | Total info | -2≤θ<-1 | -1≤θ<0 | 0≤θ<1 | 1≤θ<2 | 2≤θ<3 | Total info |
| Item 1 | 0.05 | 1.91 | 4.16 | 4.89 | 2.46 | 13.55 | 0.09 | 2.23 | 3.50 | 4.84 | 1.79 | 12.50 |
| Item 2 | 0.20 | 2.32 | 3.24 | 3.60 | 1.67 | 11.14 | 0.30 | 2.10 | 2.56 | 2.99 | 1.66 | 9.77 |
| Item 3 | 0.18 | 1.89 | 2.85 | 3.13 | 1.56 | 9.74 | 0.25 | 1.77 | 2.39 | 2.67 | 1.23 | 8.45 |
| Item 4 | 0.07 | 0.83 | 2.26 | 2.38 | 1.33 | 7.05 | 0.10 | 1.08 | 2.30 | 2.59 | 1.28 | 7.49 |
| Item 5 | 0.02 | 0.64 | 3.61 | 4.15 | 1.56 | 10.04 | 0.01 | 0.71 | 4.80 | 5.92 | 2.04 | 13.52 |
| Item 6 | 0.25 | 1.09 | 1.54 | 1.57 | 1.10 | 5.87 | 0.25 | 1.14 | 1.54 | 1.66 | 1.39 | 6.50 |
| Item 7 | 0.01 | 0.29 | 2.41 | 3.42 | 2.20 | 8.55 | 0.01 | 0.31 | 2.71 | 3.79 | 2.70 | 9.77 |
| Item 8 | 0.01 | 0.39 | 2.99 | 3.74 | 2.41 | 9.76 | 0.01 | 0.35 | 3.31 | 4.43 | 2.44 | 10.67 |
| Item 9 | 0.05 | 0.77 | 2.37 | 2.67 | 1.44 | 7.47 | 0.06 | 1.01 | 2.75 | 3.22 | 1.70 | 8.87 |
| Item 10&11 | 0.30 | 0.58 | 0.68 | 0.70 | 0.57 | 3.32 | 0.33 | 0.54 | 0.59 | 0.61 | 0.55 | 3.26 |
| Item 12 | 0.27 | 0.63 | 0.79 | 0.79 | 0.53 | 3.34 | 0.28 | 0.60 | 0.73 | 0.74 | 0.56 | 3.31 |
| Total Item | 1.42 | 11.35 | 26.92 | 31.03 | 16.83 | 89.84 | 1.68 | 11.83 | 27.19 | 33.45 | 17.34 | 94.11 |

**Supplementary Table 2. Test information for each depression scale**

|  | MHS:D Paper-pencil version | MHS:D Online version | BDI-II | CES-D | PHQ-9 |
| --- | --- | --- | --- | --- | --- |
| Test Information | 89.84 | 94.11 | 105.50 | 98.04 | 53.75 |
| Number of Items | 12 | 12 | 21 | 20 | 9 |
| Average information per item | 7.49 | 7.84 | 5.02 | 4.90 | 5.97 |


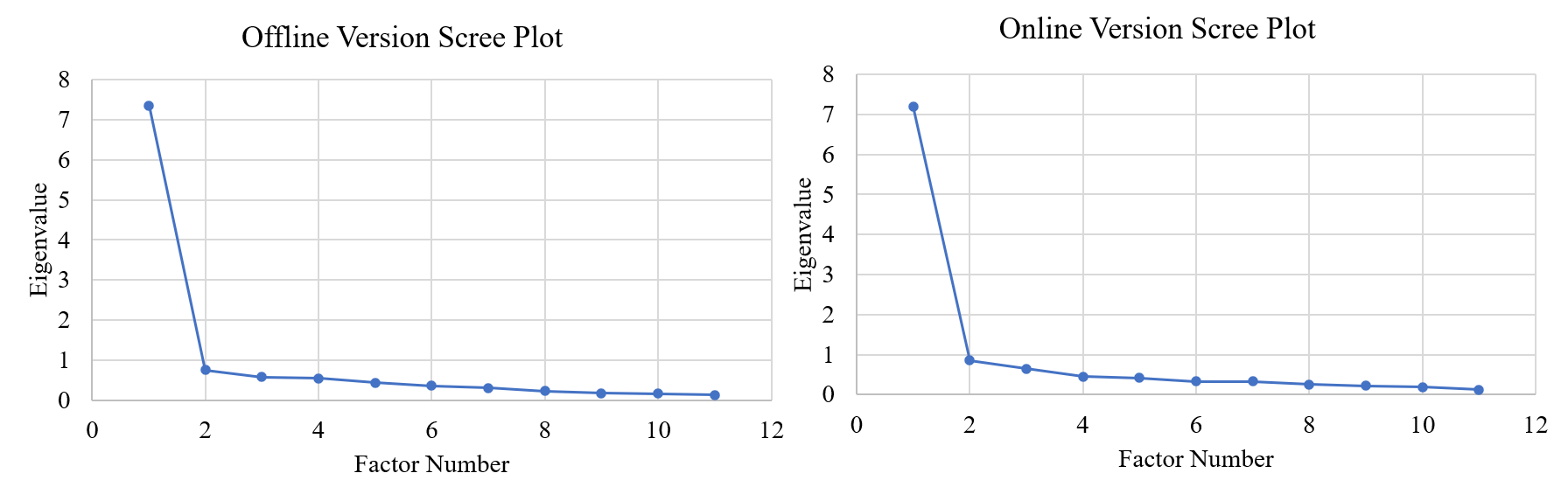


Supplementary Figure 1. Scree Plot of MHS: D


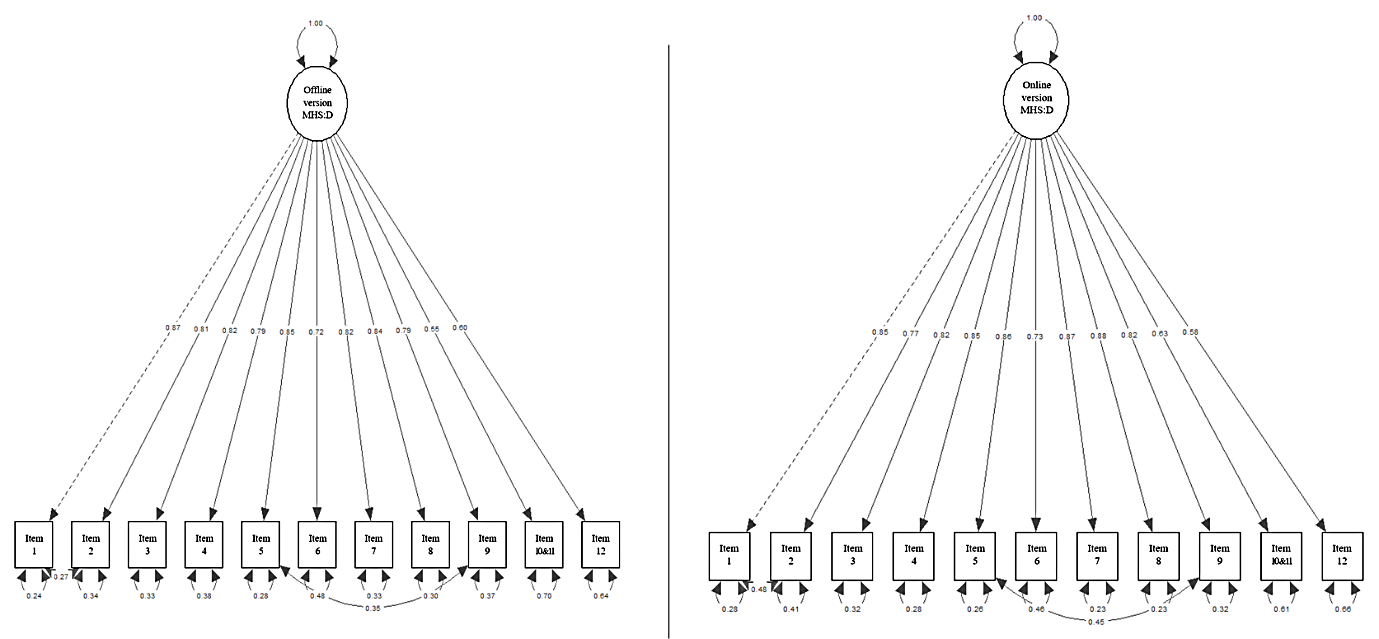


Supplementary Figure 2. Offline and Online version of MHS: D CFA 1 factor model plot

| 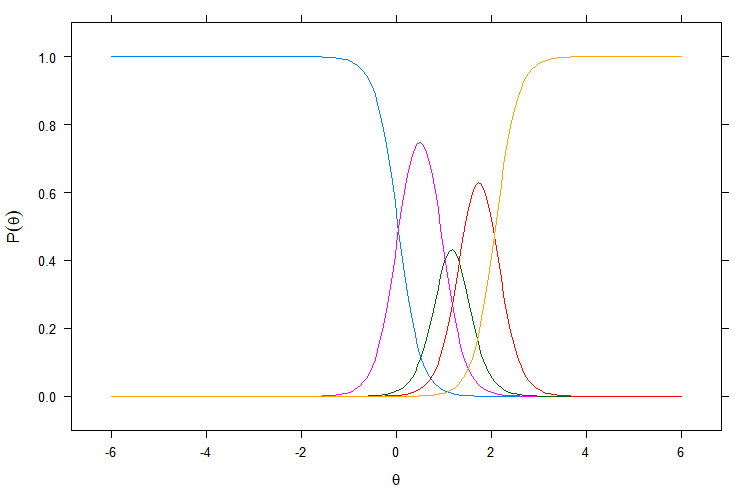 | 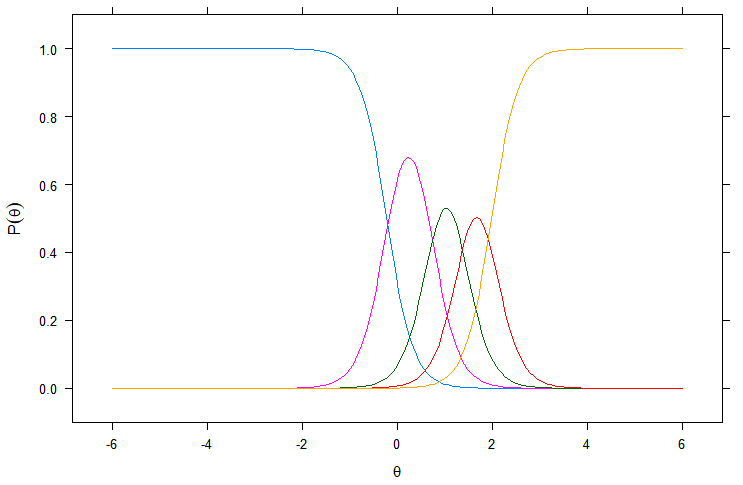 |
| --- | --- |
| Item 1 ‘Depressed Mood’ | Item 2 ‘Loss of Interest’ |
| 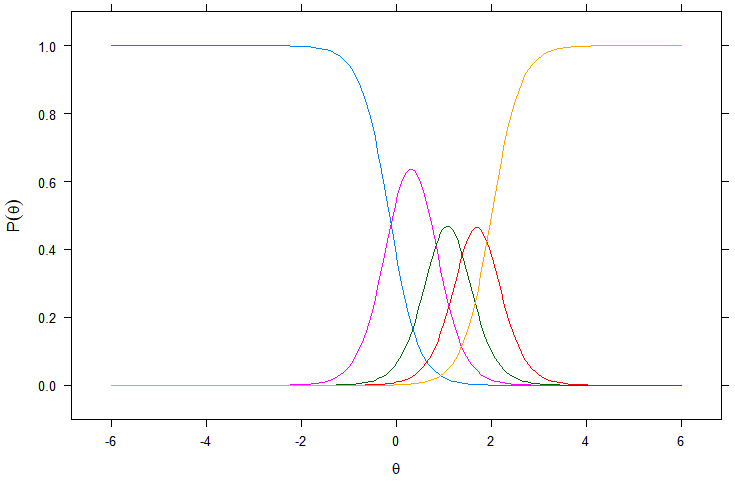 | 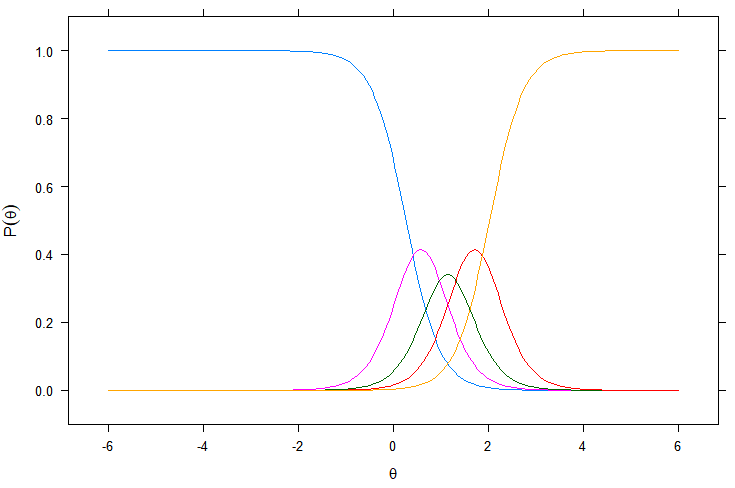 |
| Item 3 ‘Psychomotor Agitation’ | Item 4 ‘Fatigue’ |
| 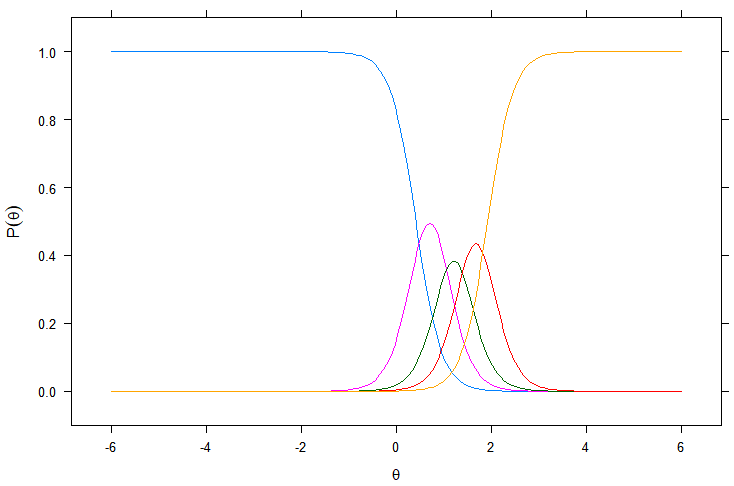 | 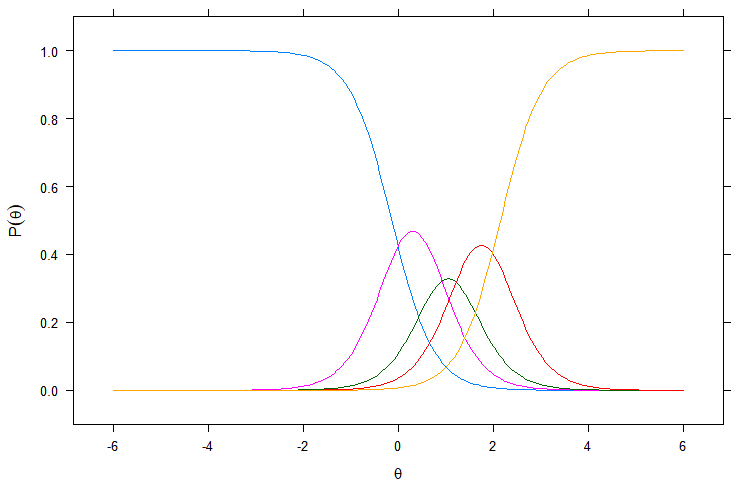 |
| Item 5 ‘Feeling worthless’ | Item 6 ‘Concentration difficulty’ |
| 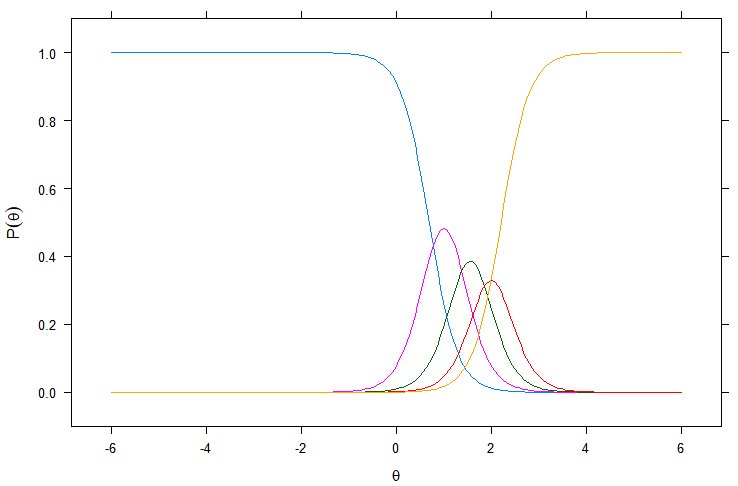 | 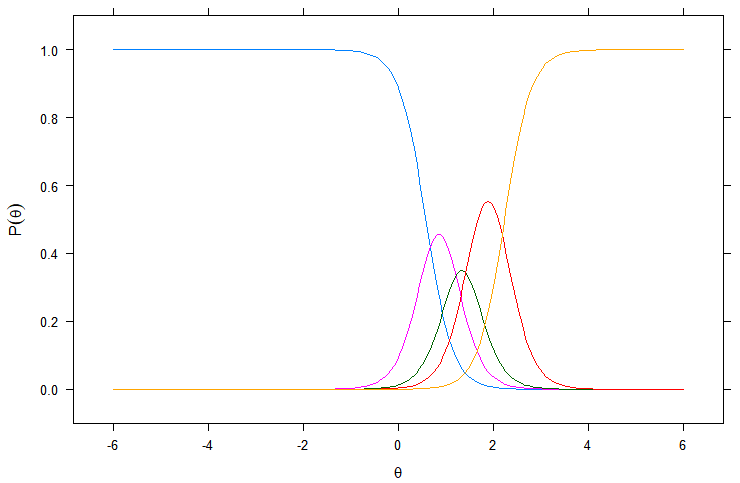 |
| Item 7 ‘Thoughts of Suicide’ | Item 8 ‘Helplessness’ |
| 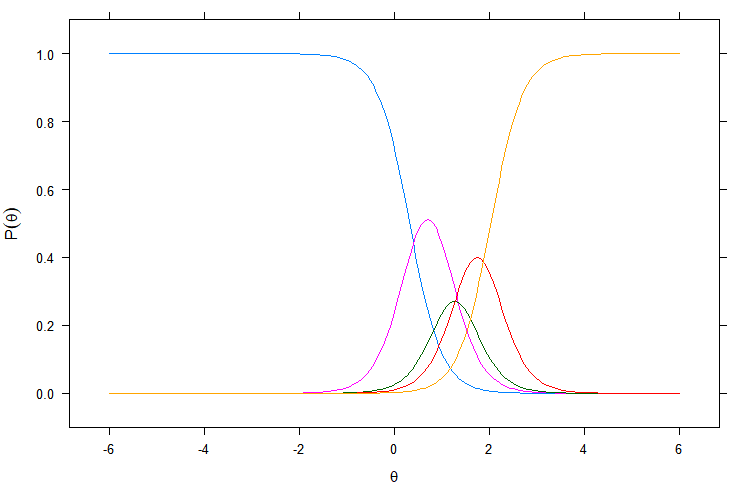 | 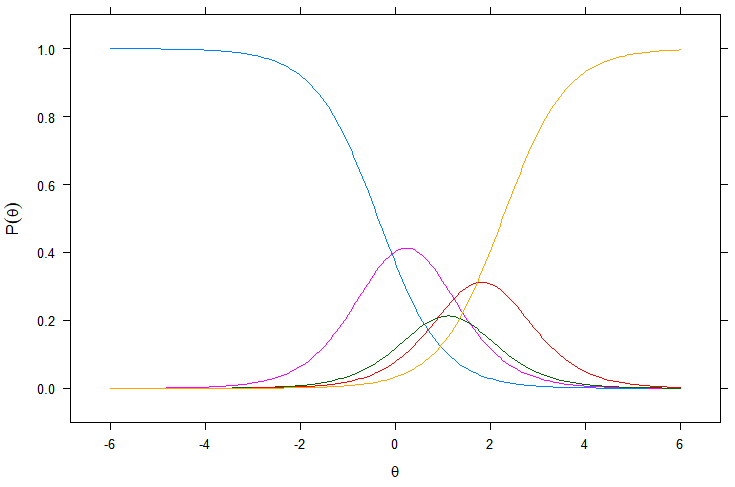 |
| Item 9 ‘Hopelessness’ | Item 10&11 ‘Increased/Decreased Appetite’ |
| 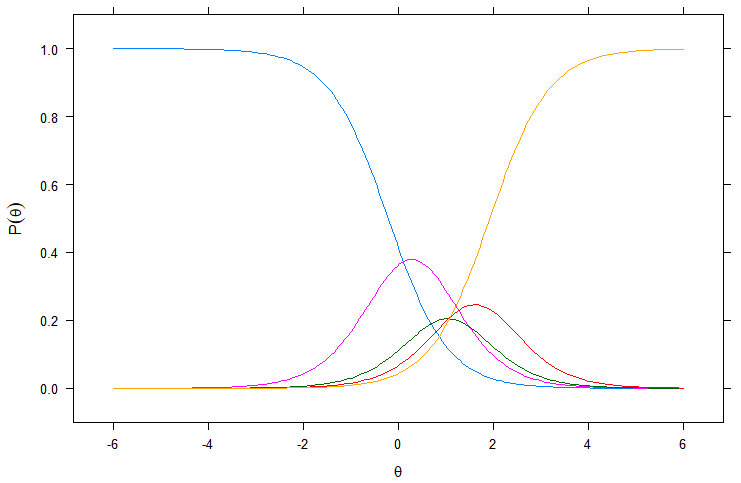 | 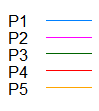 |
| Item 12 ‘Sleep Disturbance’ |  |

Supplementary Figure 3. Item Characteristic Curves of Paper-Pencil Version of MHS:D

| 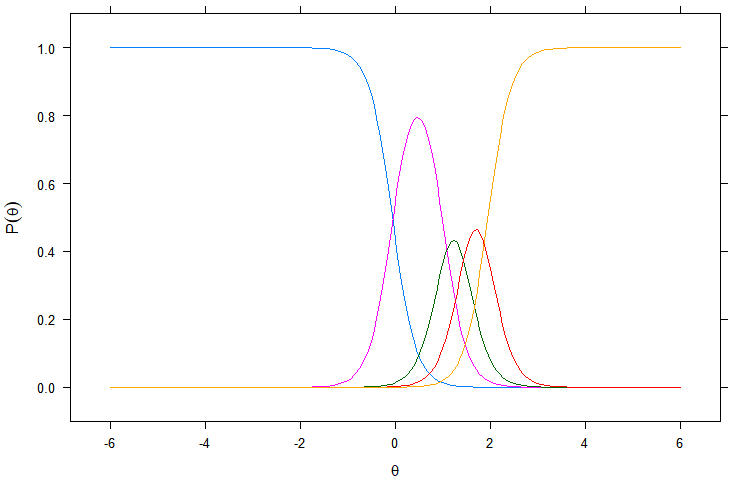 | 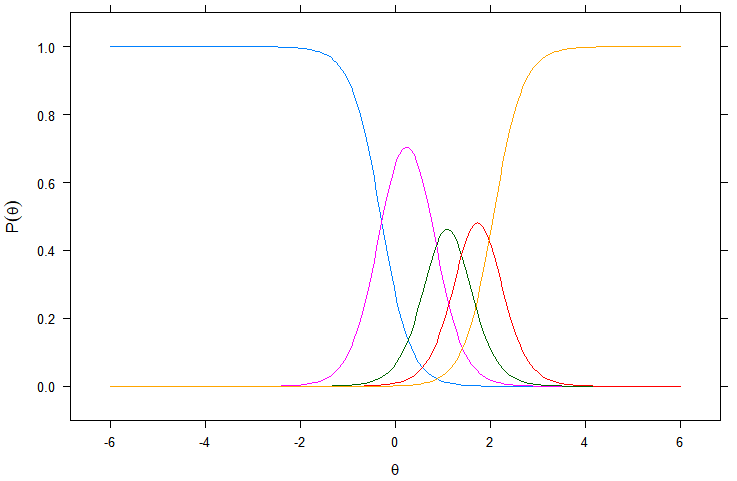 |
| --- | --- |
| Item 1 ‘Depressed Mood’ | Item 2 ‘Loss of Interest’ |
| 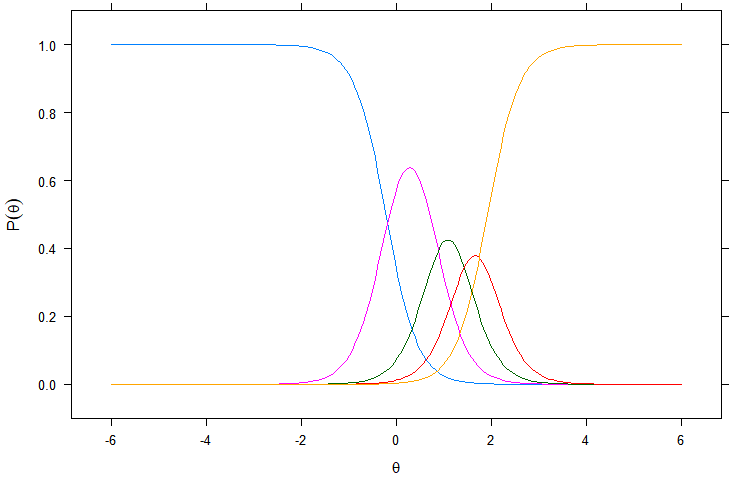 | 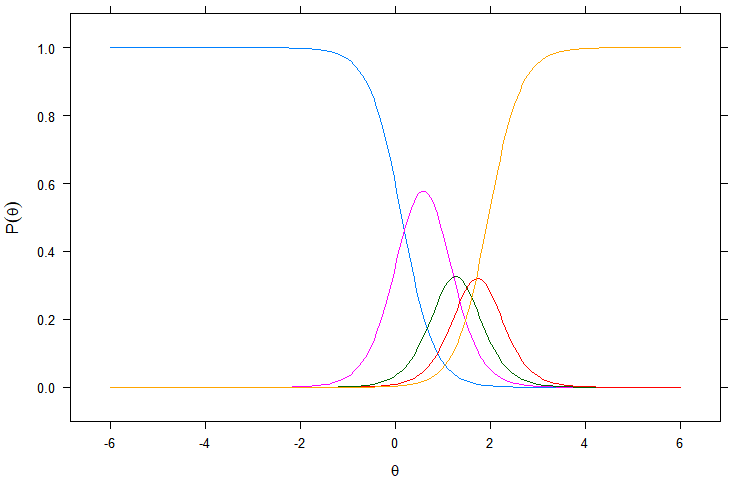 |
| Item 3 ‘Psychomotor Agitation’ | Item 4 ‘Fatigue’ |
| 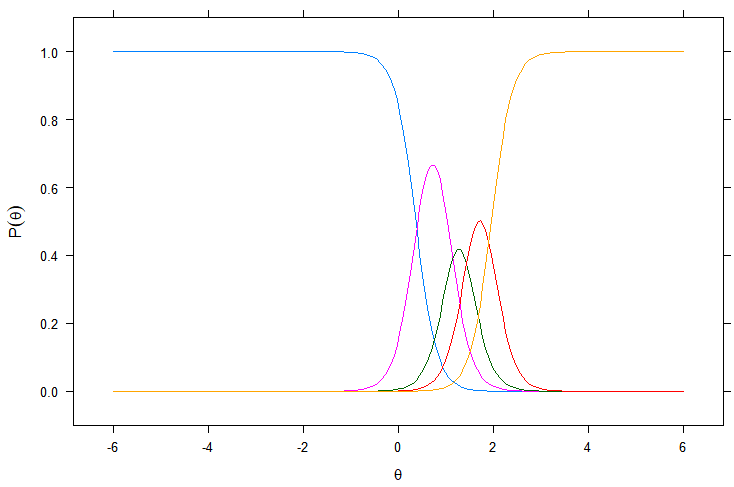 | 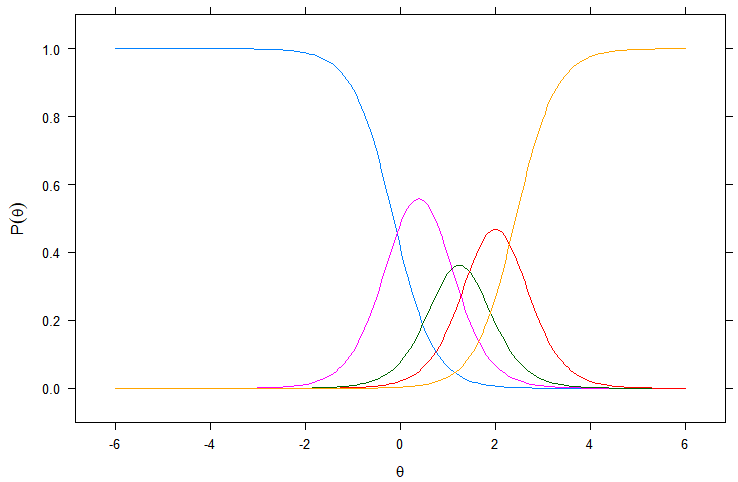 |
| Item 5 ‘Feeling worthless’ | Item 6 ‘Concentration difficulty’ |
| 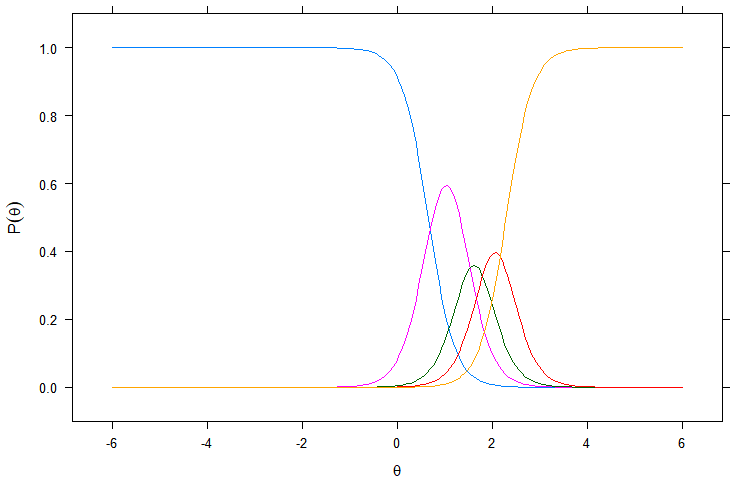 | 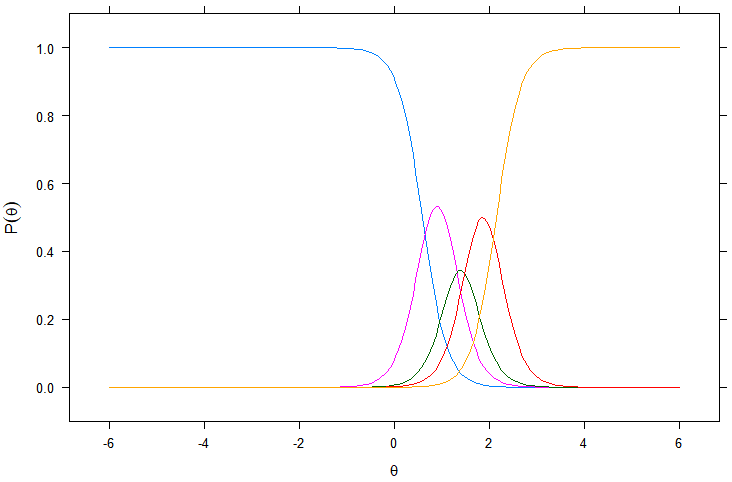 |
| Item 7 ‘Thoughts of Suicide’ | Item 8 ‘Helplessness’ |
| 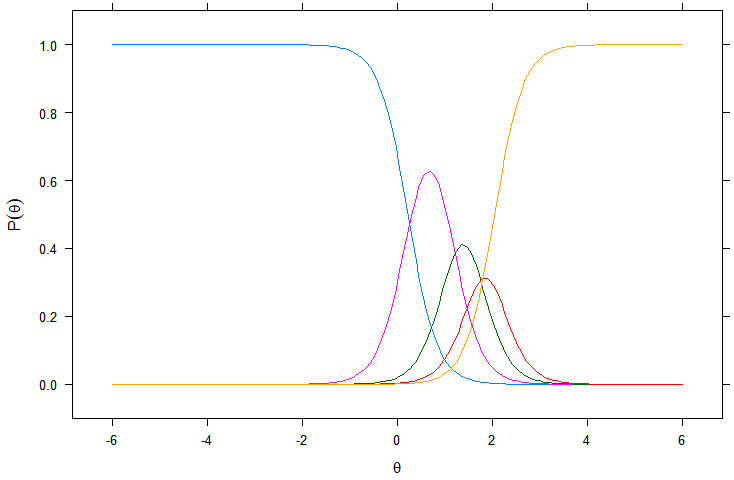 | 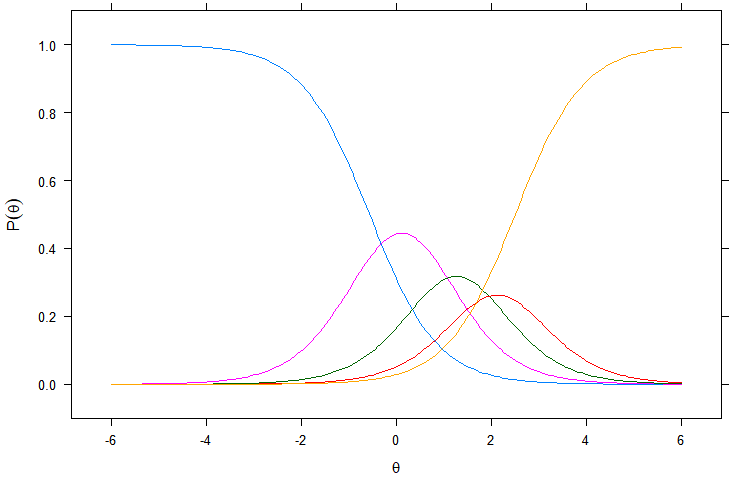 |
| Item 9 ‘Hopelessness’ | Item 10&11 ‘Increased/Decreased Appetite’ |
| 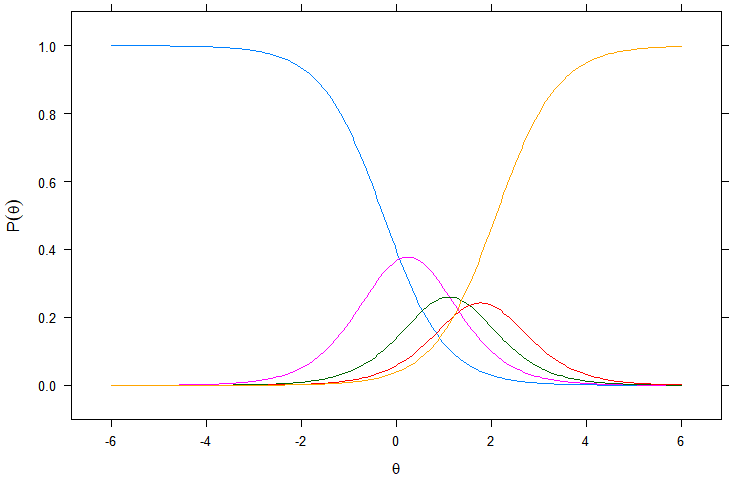 | 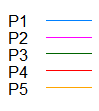 |
| Item 12 ‘Sleep Disturbance’ |  |

Supplementary Figure 4. Item Characteristic Curves of Online Version of MHS:D

| 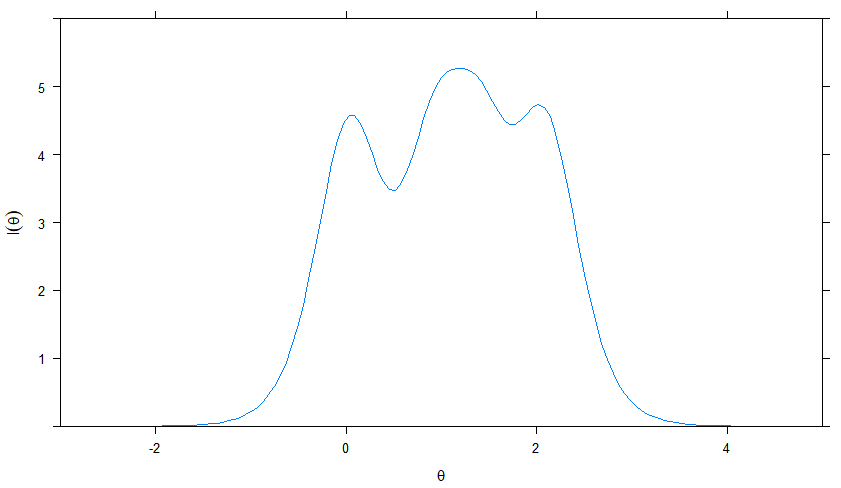 | 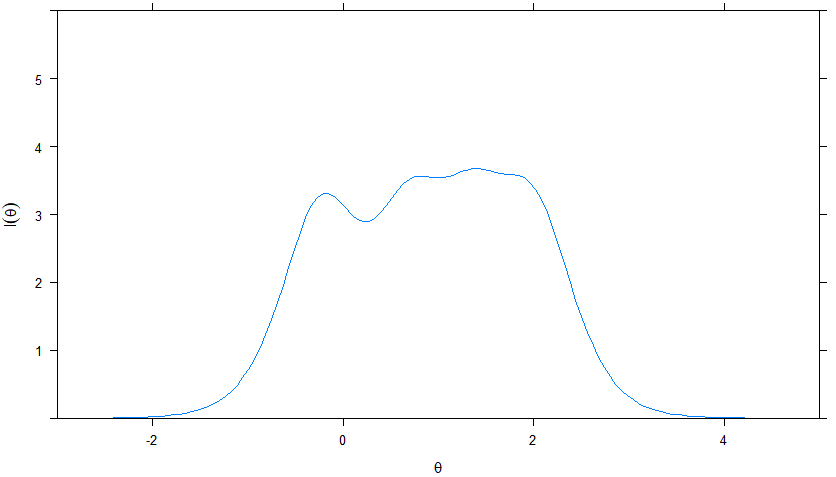 |
| --- | --- |
| Item 1 ‘Depressed Mood’ | Item 2 ‘Loss of Interest’ |
| 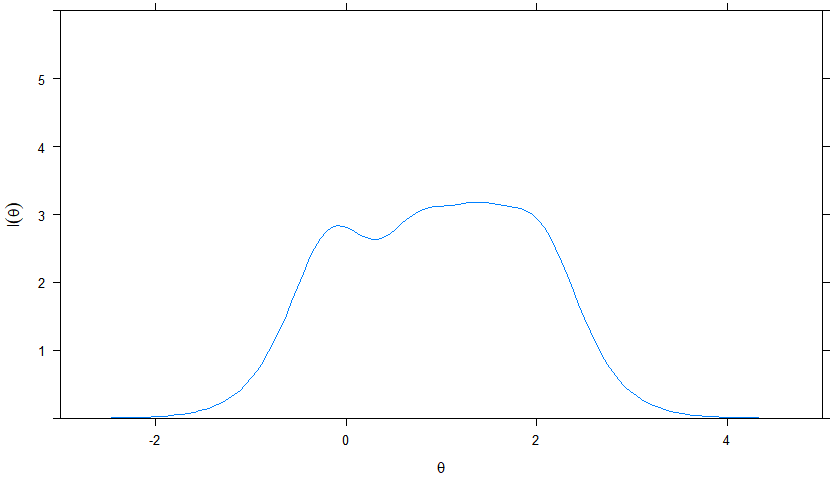 | 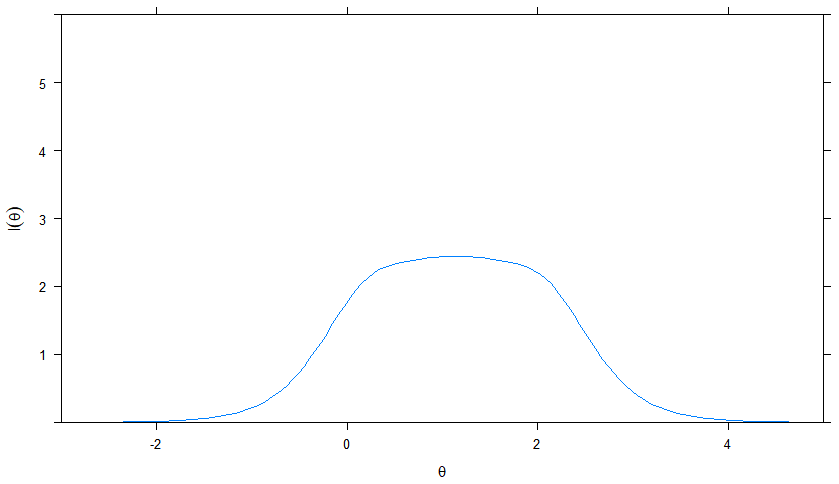 |
| Item 3 ‘Psychomotor Agitation’ | Item 4 ‘Fatigue’ |
| 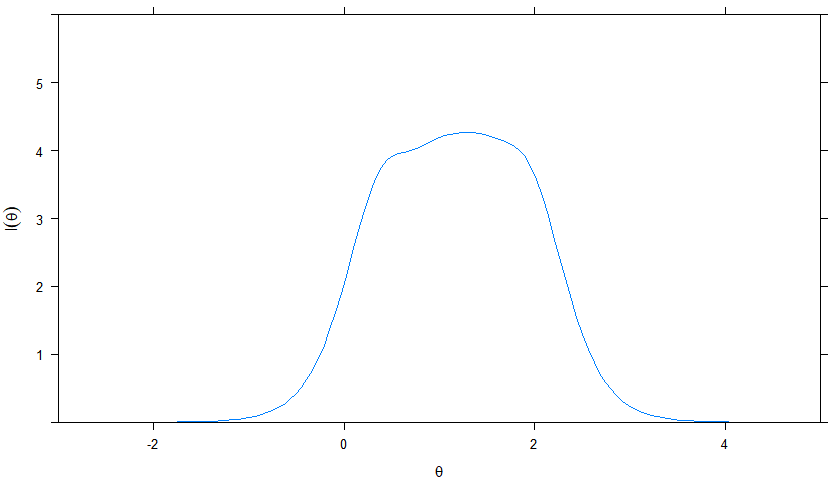 | 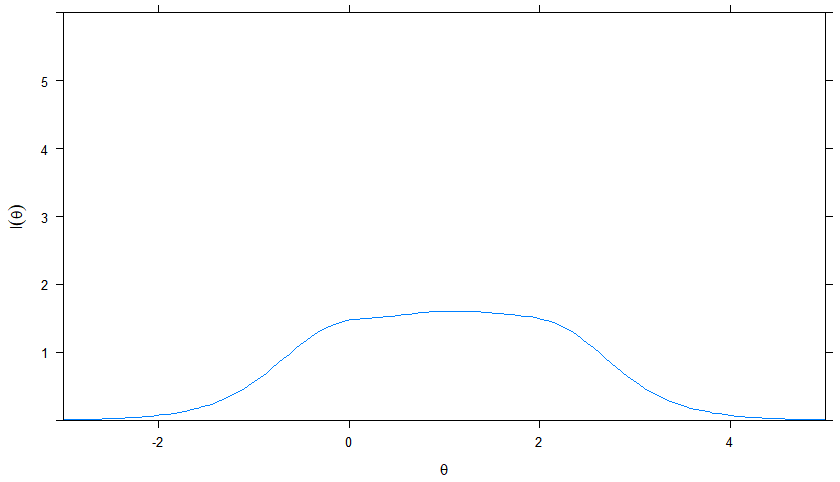 |
| Item 5 ‘Feeling worthless’ | Item 6 ‘Concentration difficulty’ |
| 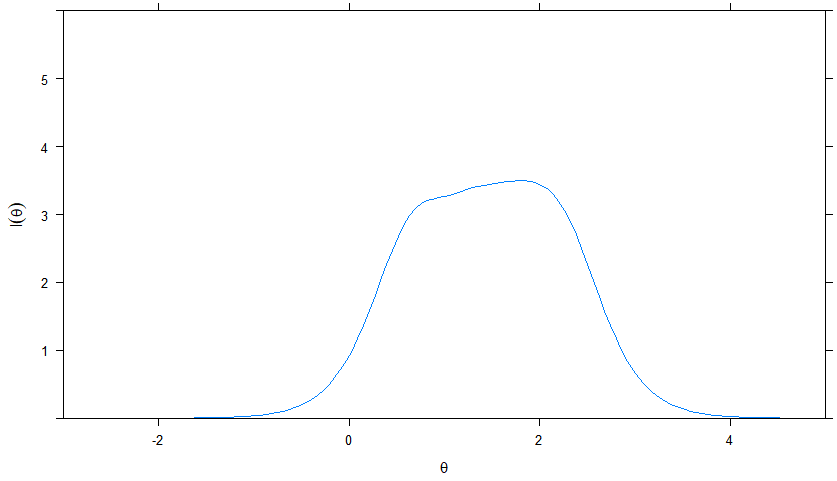 | 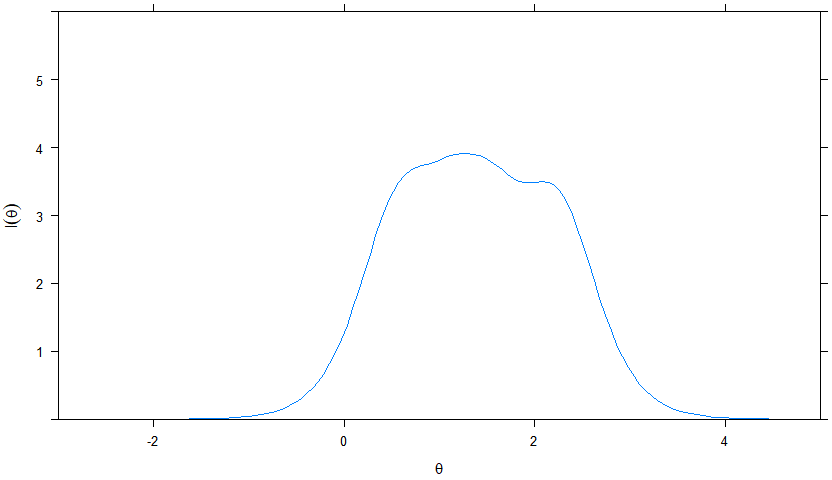 |
| Item 7 ‘Thoughts of Suicide’ | Item 8 ‘Helplessness’ |
| 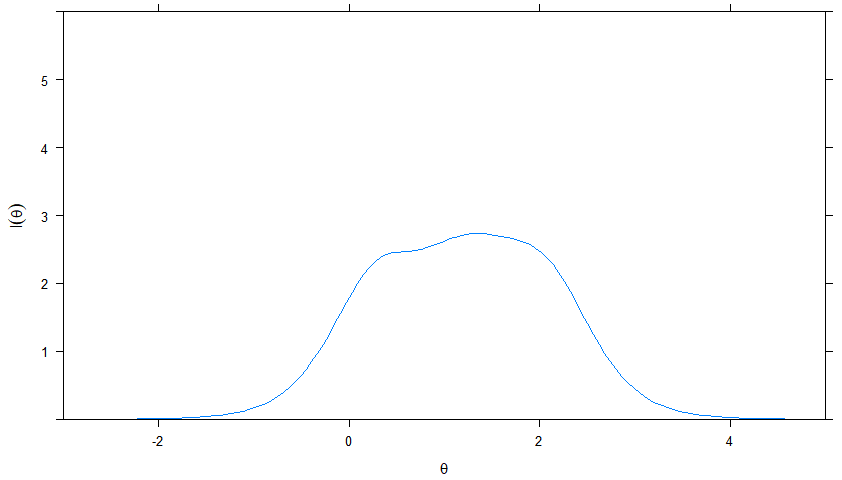 | 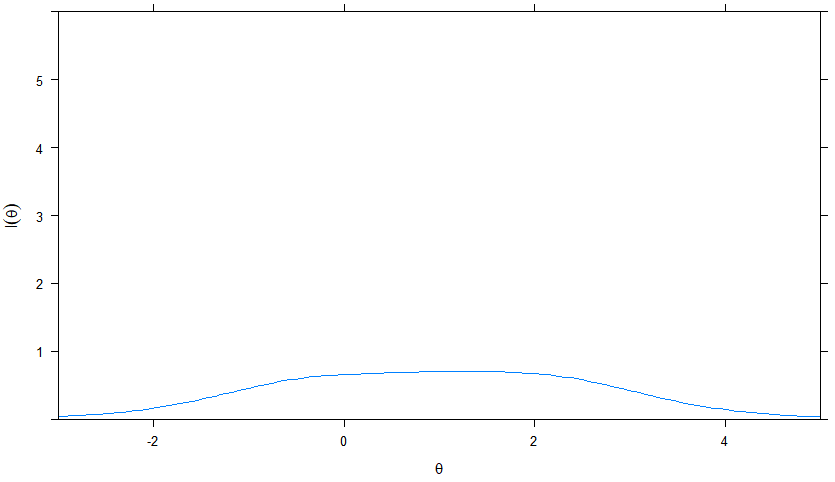 |
| Item 9 ‘Hopelessness’ | Item 10&11 ‘Increased/Decreased Appetite’ |
| 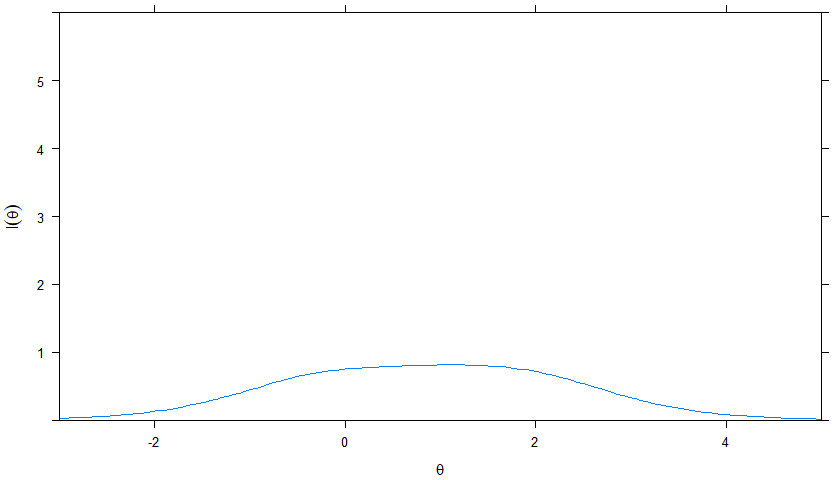 |  |
| Item 12 ‘Sleep Disturbance’ |  |

Supplementary Figure 5. Item Information Curves of Paper-Pencil Version of MHS:D

| 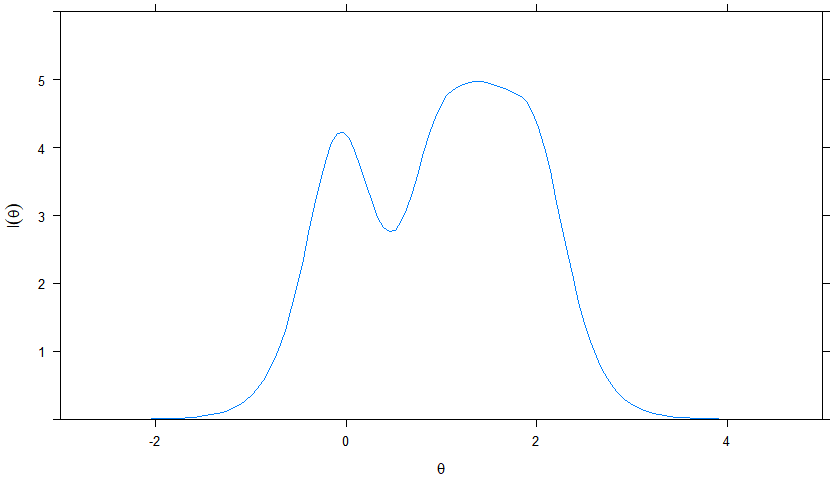 | 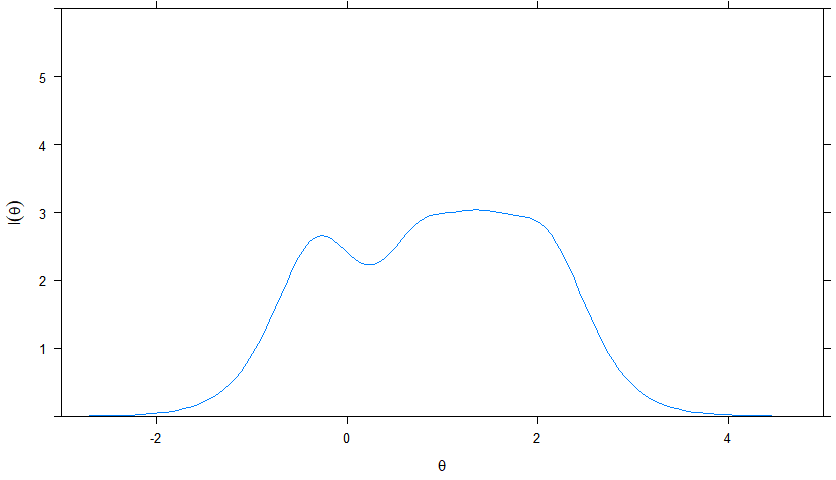 |
| --- | --- |
| Item 1 ‘Depressed Mood’ | Item 2 ‘Loss of Interest’ |
| 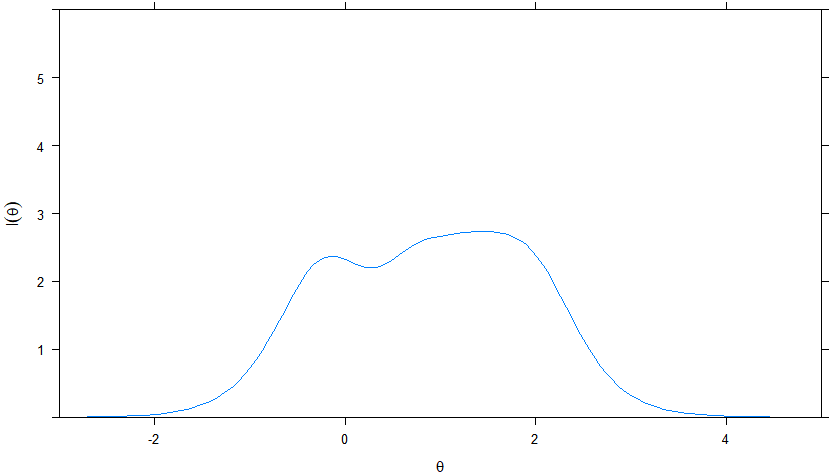 | 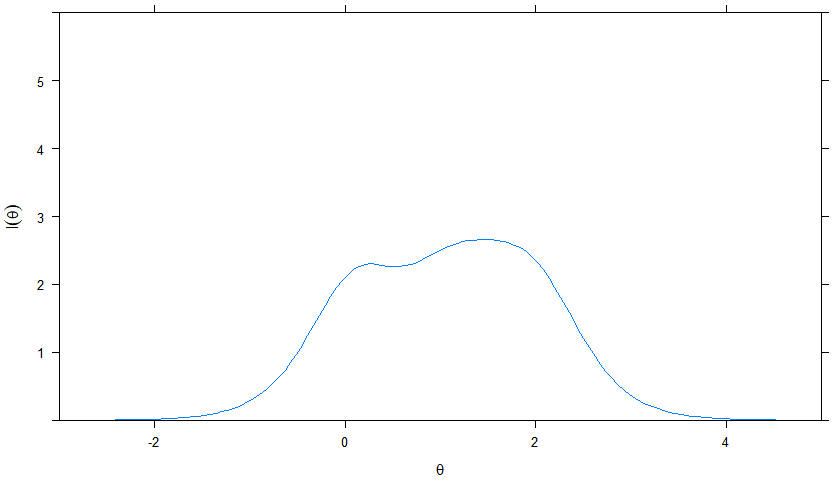 |
| Item 3 ‘Psychomotor Agitation’ | Item 4 ‘Fatigue’ |
| 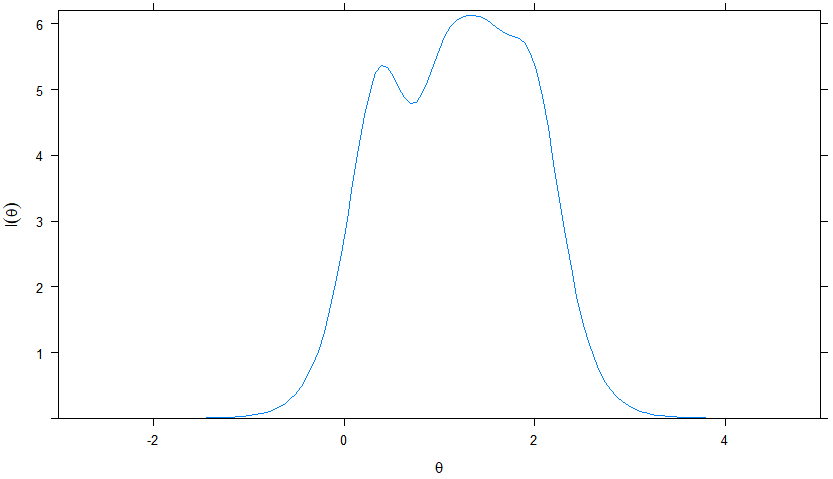 | 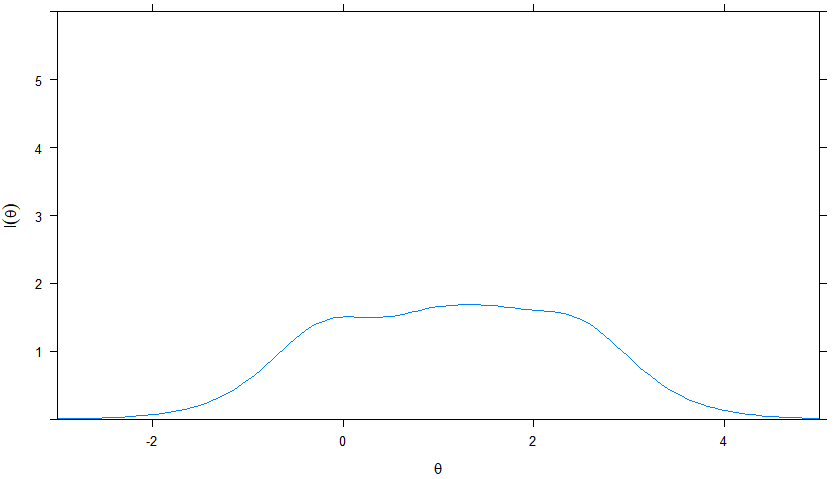 |
| Item 5 ‘Feeling worthless’ | Item 6 ‘Concentration difficulty’ |
| 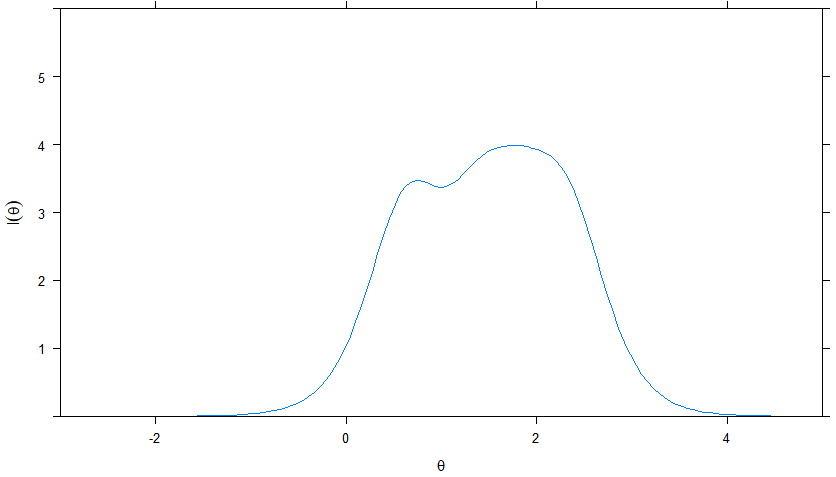 | 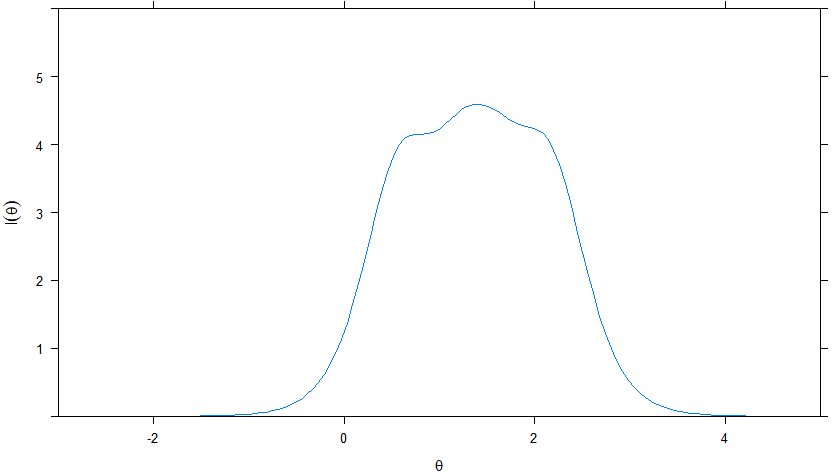 |
| Item 7 ‘Thoughts of Suicide’ | Item 8 ‘Helplessness’ |
| 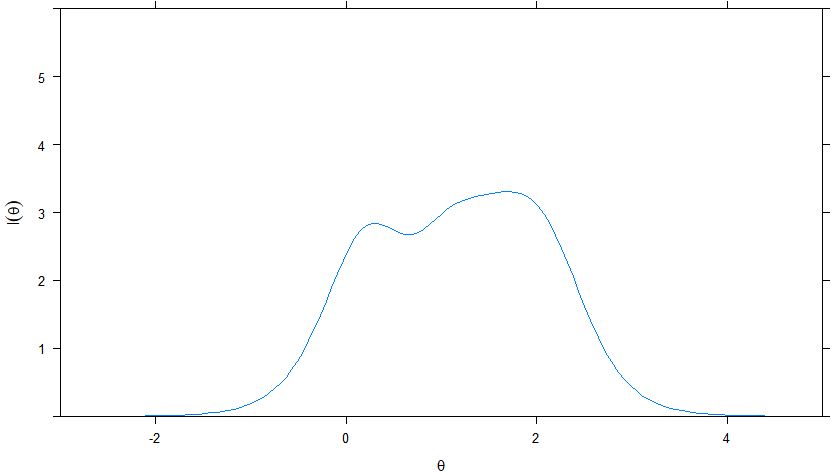 | 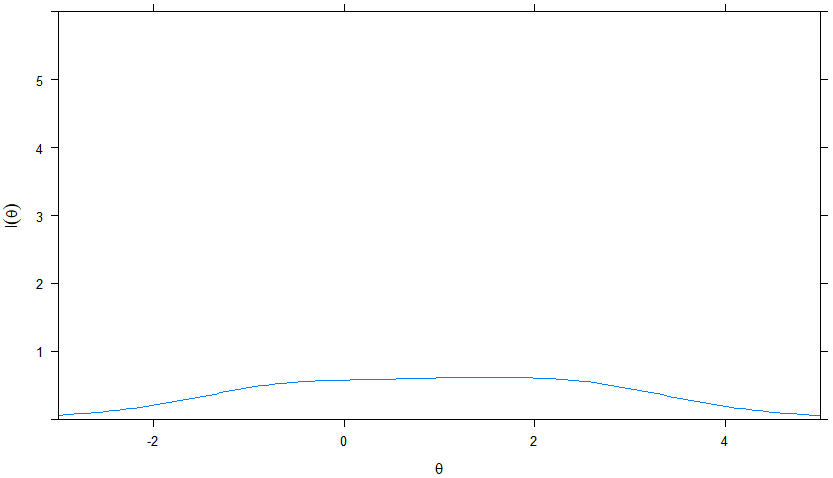 |
| Item 9 ‘Hopelessness’ | Item 10&11 ‘Increased/Decreased Appetite’ |
| 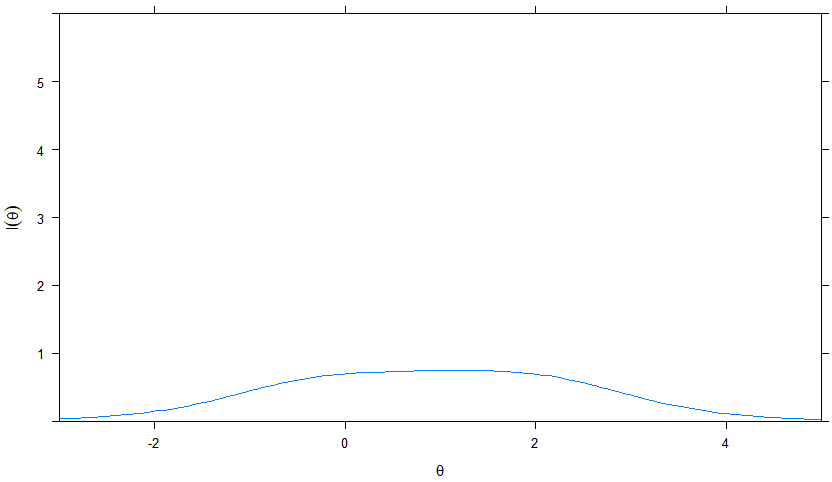 |  |
| Item 12 ‘Sleep Disturbance’ |  |

Supplementary Figure 6. Item Information Curves of Online Version of MHS:D


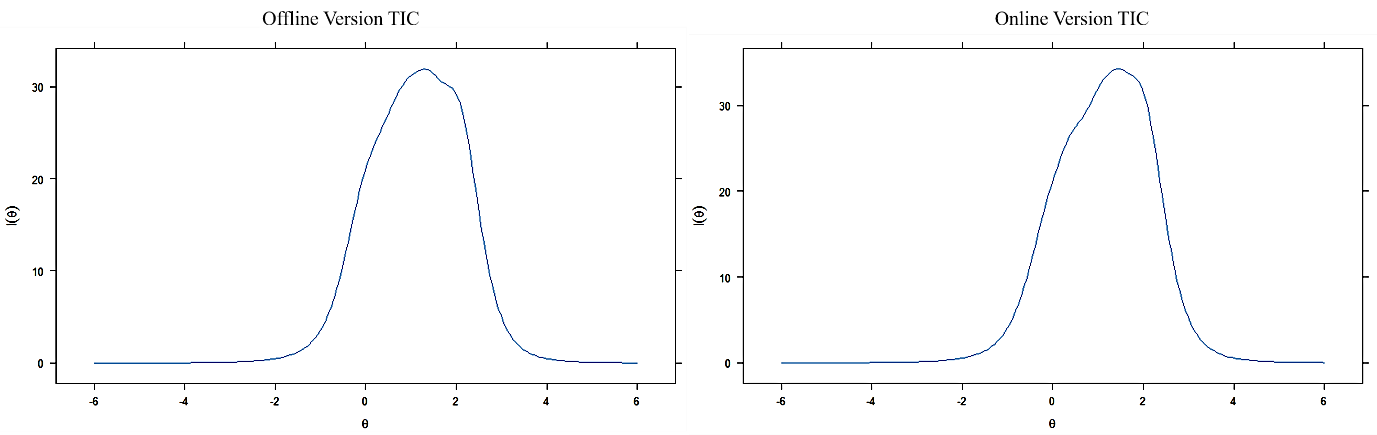
**Supplementary Figure 7. Test Information Curve for MHS: D**
